# Phylogeny, divergence time and historical biogeography of *Phanerochaete* (*Basidiomycota*, *Polyporales*), with a description of two new species in Korea

**DOI:** 10.3897/imafungus.17.204198

**Published:** 2026-08-20

**Authors:** Minseo Cho, Sun Lul Kwon, Yeonjae Yoo, Sang Hyun Lee, Changmu Kim, Young Woon Lim, Jae-Jin Kim

**Affiliations:** 1 Division of Environmental Science and Ecological Engineering, College of Life Sciences and Biotechnology, Korea University, Seoul, Republic of Korea Biological Conservation Research Division, National Institute of Biological Resources Incheon Republic of Korea; 2 Division of Wood Engineering, National Institute of Forest Science, Seoul, Republic of Korea Division of Environmental Science and Ecological Engineering, College of Life Sciences and Biotechnology, Korea University Seoul Republic of Korea; 3 Biological Conservation Research Division, National Institute of Biological Resources, Incheon, Republic of Korea School of Biological Sciences and Institute of Biodiversity, Seoul National University Seoul Republic of Korea; 4 School of Biological Sciences and Institute of Biodiversity, Seoul National University, Seoul, Republic of Korea Division of Wood Engineering, National Institute of Forest Science Seoul Republic of Korea

**Keywords:** Corticioid fungi, evolutionary analysis, multi-locus phylogeny, novel species, *
Phanerochaetaceae
*

## Abstract

The genus *Phanerochaete* (*Basidiomycota*, *Polyporales*) is a member of corticioid fungi. Recent phylogenetic studies have reclassified *Phanerochaete* species into other genera and delimited the core *Phanerochaete*. However, phylogenetic studies using multi-locus datasets comprising nuclear ribosomal internal transcribed spacer (ITS), nuclear large subunit ribosomal DNA (nLSU), translation elongation factor 1–α (*tef1*), RNA polymerase II largest subunit (*rpb1*) and RNA polymerase II second largest subunit (*rpb2*) are still limited and the divergence time estimation and historical biogeography are under-represented for the genus *Phanerochaete*. To address this gap, we conducted the first integrative evolutionary study of *Phanerochaete* using a five-locus dataset. Of the 72 specimens, 14 species were identified, including two novel species: *Phanerochaete koreana***sp. nov**., characterised by coriaceous basidiome and the absence of leptocystidia and *Phanerochaete membranacea***sp. nov**., characterised by an ochraceous membranaceous basidiomata and heavily encrusted leptocystidia. Multi-locus phylogenetic analysis, divergence time estimation and historical biogeographic analyses suggest that *Phanerochaete* originated during the Early Cretaceous, with a mean crown age of 101.66 Mya [95% highest posterior density (HPD): 98.38–104.86 Mya]. This study elucidates the evolutionary relationships between *Phanerochaete* and allied genera within *Polyporales* and explains the global diversity, phylogeny and evolution of the genus.

## Introduction

*Phanerochaete* P. Karst was introduced by Karsten in 1889 and is typified by *P. alnea* (Fr.) P. Karst (Karsten 1889). *Phanerochaete* species are characterised by effused, membranaceous and crustaceous basidiomata with smooth hymenial surfaces, although a few have hydnoid, odontioid or raduloid forms (Eriksson et al. 1978; Burdsall Jr 1985; Floudas and Hibbett 2015; Volobuev et al. 2015; Miettinen et al. 2016; Chen et al. 2021; Larsson et al. 2025). They have a monomitic hyphal system without clamp connections, cylindrical cystidia and ellipsoid to cylindrical basidiospores. *Phanerochaete* is one of the diverse genera in the family *Phanerochaetaceae (Polyporales)*, that causes white rot on the twigs, logs, stumps and fallen branches of both angiosperms and gymnosperms (Floudas and Hibbett 2015). As white-rot fungi, *Phanerochaete* species produce ligninolytic and cellulolytic enzymes. The enzymatic activity of *P. chrysosporium* has been extensively studied; it produces industrially significant enzymes, such as lignin peroxidases, manganese peroxidases and glyoxal oxidases (Tien and Kirk 1983, 1984; Kirk et al. 1986; Kersten and Kirk 1987). Owing to their strong lignocellulose degrading capabilities, the whole genomes of *P. carnosa* and *P. chrysosporium* have been sequenced and these species have been used as model species to study wood degradation mechanisms (Martinez et al. 2004; Kersten and Cullen 2007; Suzuki et al. 2012).

During the 20^th^ century, many *Phanerochaete*-like corticioid species were broadly assigned to *Phanerochaete*. Several studies have highlighted the need to delimit this genus. Based on morphological characteristics, such as the presence of cystidia and the development of the subiculum, new genera, *Phanericium* and *Scopuloides*, have been proposed (Parmasto 1968; Eriksson et al. 1978; Burdsall Jr 1985). Advancements in DNA-based molecular analyses have indicated that *Phanerochaete* is polyphyletic and nested across multiple lineages in three different families (*Irpicaceae*, *Meruliaceae* and *Phanerochaetaceae*), which is considered as phlebioid clade (De Koker et al. 2003; Wu et al. 2010; Floudas and Hibbett 2015). This finding emphasises the need for the phylogenetic delimitation of *Phanerochaete* sensu lato. Subsequently, molecular phylogenetic studies, primarily based on ITS and nLSU sequence data, indicated that the core species are now recognised as *Phanerochaete* sensu stricto, whereas others have been re-assigned to different genera in the phlebioid clade, including *Ceriporia*, *Efibula*, *Hydnophanerochaete*, *Hydnophlebia*, *Luteochaete*, *Phaeophlebiopsis*, *Phanericium*, *Phanerochaetella*, *Phaneroites*, *Phlebiopsis*, *Rhizochaete* and *Scopuloides* (Greslebin et al. 2004; Zmitrovich et al. 2006; Hjortstam and Ryvarden 2010; Floudas and Hibbett 2015; Chikowski et al. 2016; Chen et al. 2018b; Chen et al. 2021; Wang et al. 2023; Larsson et al. 2025).

According to the MycoBank database (http://www.MycoBank.org) and the Species Fungorum (http://www.speciesfungorum.org) (accessed 13 July 2026), 212 species (excluding infraspecific taxa) are listed under *Phanerochaete*, of which 127 are currently accepted. Of the 127 accepted species, sequence data were available for 87 species, whereas the remaining 40 species were described solely based on morphological characteristics and lacked molecular data. Therefore, *Phanerochaete* species lacking DNA barcode sequences require further phylogenetic assessment using multi-locus datasets, particularly following recent taxonomic revisions.

Recent phylogenetic studies on *Phanerochaete* have primarily focused on species diversity, taxonomic revision and species identification, based on ITS and nLSU sequences (Xu et al. 2020; Li et al. 2023; Zhang et al. 2023; Luo et al. 2024; Xu et al. 2025). However, multi-locus phylogenetic studies incorporating protein-coding markers such as *tef1*, *rpb1* and *rpb2* remain limited. Recent systematic studies on *Polyporales* have clarified the phylogenetic relationships of numerous polyphyletic groups and species complexes (Miettinen et al. 2016; Justo et al. 2017; Chen et al. 2021; Ji et al. 2023; Liu et al. 2023b). At the genus level, only a few genera within *Polyporales*, including *Hyphoderma*, *Laetiporus* and *Phaeolus* have been comprehensively studied in phylogeny, divergence time and biogeography (Song and Cui 2017; Cui et al. 2026; Li et al. 2026). However, comparable integrative evolutionary studies remain lacking in *Phanerochaete*. Therefore, integrative analyses, combining multi-locus phylogeny, divergence time estimation and historical biogeography, are necessary to understand the taxonomy, evolutionary relationships and diversification history of the genus.

Divergence time estimation incorporates the molecular evolutionary rates and fossil calibrations to infer the geological ages of fungal lineages, thereby strengthening phylogenetic interpretations (Zhao et al. 2016; Zhao et al. 2017; He et al. 2019; Varga et al. 2019; Ji et al. 2022). Likewise, genus-level biogeographical analyses have provided insights into the origin, diversification and current distribution patterns (Chen et al. 2015; Song et al. 2016; Song and Cui 2017; Zhu et al. 2019; Zhao et al. 2022; Zhao et al. 2025; Cui et al. 2026; Li et al. 2026). Within the phlebioid clade of *Polyporales*, divergence time analyses have been conducted for the families *Irpicaceae* and *Meruliaceae* (Li et al. 2025a; Wang et al. 2025a). Accordingly, comprehensive studies integrating morphology, multilocus phylogeny, divergence time estimation and historical biogeography are still needed to understand *Phanerochaete*.

This study aimed to clarify the taxonomic framework, evolutionary relationships and diversification history of *Phanerochaete* through integrative analyses, based on multi-locus phylogeny, divergence time estimation and historical biogeography. To achieve this, newly-generated sequences from Asian specimens, including two newly-described species from Korea, were analysed together with worldwide representative *Phanerochaete* sequences, including type-derived sequences. Morphological observations and multi-locus phylogenetic analyses were used to provide a comprehensive framework for understanding the taxonomy, diversification and evolutionary history of *Phanerochaete*.

## Materials and methods

### Specimen collection

A total of 72 specimens were collected throughout the Republic of Korea between 2010 and 2024. Collected samples were dried at 55 °C for 72 h and stored with silica gel in a paper bag under dry conditions. The specimens were deposited at the Korea University Culture Collection (**KUC**), Seoul National University Fungus Collection (**SFC**) and the National Institute of Biological Resources (**NIBR**).

### Morphological observations

Macromorphological images of each specimen were captured using a Sony ɑ 6500 camera (Sony, Tokyo, Japan). The size, shape, texture and colour of the basidiomata were recorded. Micromorphological structures, including basidia, basidiospores, hyphae and leptocystidia, were observed and measured using an Olympus BX51 light microscope (Olympus, Tokyo, Japan) at 40–1000× magnification after mounting in 5% potassium hydroxide (KOH). Micrographs were captured using a DP20 camera (Olympus, Tokyo, Japan). At least 20 basidia, basidiospores and leptocystidia were observed. Colour descriptions followed the Munsell Soil Colour Book (Munsell 2009). The following abbreviations are used: L = mean basidiospore length; W = mean basidiospore width; Q = length/width ratio; *x* = number of basidiospores measured; *y* = number of specimens; and n = *x*/*y*. Reactions of hyphae and basidiospores to Cotton Blue (CB) and Melzer’s reagent (IKI) and 5% KOH were examined. CB– indicate acyanophilous, IKI– indicates neither amyloid nor dextrinoid and KOH reactions were assessed, based on changes in the hyphal tissues.

### DNA extraction, amplification and sequencing

Genomic DNA was extracted from dried specimens using an AccuPrep Genomic DNA Extraction Kit (Bioneer, Daejeon, Korea) following the manufacturer’s instructions. The nuclear ribosomal internal transcribed spacer (ITS) region was amplified using the primer pairs ITS5/ITS4 or ITS5/LR3 (Vilgalys and Hester 1990; White et al. 1990). The nuclear large subunit ribosomal DNA (nLSU) region was amplified using LR0R/LR5 (Vilgalys and Hester 1990). The translation elongation factor 1-alpha (*tef1*) region was amplified using EF1-983F/EF1-1567R (Rehner and Buckley 2005). The RNA polymerase II largest subunit (*rpb1*) region was amplified using RPB1-Af/RPB1-Cr (Matheny et al. 2002) and the RNA polymerase II second largest subunit (*rpb2*) region was amplified using fRPB2-5F/fRPB2-7CR (Matheny 2005).

PCR conditions for ITS and nLSU regions consisted of an initial denaturation at 95 °C for 5 min, followed by 35 cycles of 95 °C for 40 s, 55 °C for 40 s and 72 °C for 1 min, with a final extension at 72 °C for 5 min. For the *tef1* region, PCR conditions were an initial denaturation at 96 °C for 5 min, followed by 33 cycles of 96 °C for 1 min, 45 °C for 1 min and 72 °C for 1 min, with a final extension at 72 °C for 7 min. For the *rpb1* and *rpb2* regions, PCR amplification was performed with a two-stage cycling programme. After an initial denaturation at 94 °C for 2 min, the first stage consisted of 10 cycles of 94 °C for 40 s, 60 °C for 40 s and 72 °C for 1 min, followed by a second stage of 37 cycles of 94 °C for 45 s, 53 °C for 1.5 min and 72 °C for 2 min with a final extension at 72 °C for 10 min (Suppl. material 2). PCR products were purified using the AccuPrep PCR Purification Kit (Bioneer, Daejeon, Korea). Sanger sequencing was conducted by Cosmogenetech (Seoul, Korea). The resulting sequences were edited and assembled using Geneious Prime 2026.0.2 (https://www.geneious.com). The newly-generated sequences were deposited in GenBank (Table 1).

**Table 1.** Species information and GenBank accession numbers of *Phanerochaete* and related taxa used in this study.

**Species name**	**Specimen voucher**	**Country**	**GenBank Accession No**.					**References**
			**ITS**	**nLSU**	***tef1***	***rpb1***	***rpb2***	
*Agaricus campestris*	LAPAG370	Unknown	KM657927	KR006607	KR006636	–	KT951556	(Zhou et al. 2016a)
*Amylocorticium cebennense*	HHB-2808	USA	GU187505	GU187561	GU187675	GU187439	GU187770	(Binder et al. 2010)
*Athelia arachnoidea*	CBS 418.72	Netherlands	GU187504	GU187557	GU187672	GU187436	GU187769	(Binder et al. 2010)
*Auricularia heimue*r	Xiaoheimao	China	LT716074	KY418890	KY419083	–	KY419035	(Zhao et al. 2017)
*Bjerkandera adusta*	HHB-12826-Sp	USA: Alaska	KP134983	KP135198	KT305938	KP134784	KP134913	(Floudas and Hibbett 2015)
*Boletopsis leucomelaen*a	AFTOL-ID 1527	USA	DQ484064	DQ154112	GU187763	GU187494	GU187820	(Matheny et al. 2006)
*Boletus edulis*	HMJAU 4637	Unknown	JN563894	KF112455	KF112202	KF112586	KF112704	(Feng et al. 2012)
*Bondarzewia mesenterica*	AFTOL-ID 452	Canada	DQ200923	DQ234539	DQ059044	DQ256049	AY218474	(Matheny et al. 2007)
*Byssomerulius corium*	FCUG 2701	Russia	MZ636931	GQ470630	MZ913668	MZ748415	OK136068	(Wu et al. 2010; Chen et al. 2021)
*Ceriporiopsis subvermispora*	FD-354	USA	KP135312	KP135212	–	KP134879	–	(Floudas and Hibbett 2015)
*Cerrena unicolor*	FD-299	USA	KP135304	KP135209	–	KP134874	KP134968	(Floudas and Hibbett 2015)
*Crystallicutis rajchenbergii*	MR-4310 ^T^	USA	KY948797	KY948888	MZ913711	KY948963	OK136093	(Justo et al. 2017; Chen et al. 2021)
*Cytidiella albomarginata*	WEI 18-474 ^T^	Taiwan	MZ636948	MZ637110	MZ913678	MZ748429	OK136070	(Chen et al. 2021)
*Dacrymyces spathularia*	AFTOL-ID 454	USA	AY854070	AY701525	AY881020	AY857981	AY786054	(Lutzoni et al. 2004)
*Donkia pulcherrim*a	GC 1707-11	Taiwan	LC378994	LC379152	LC387371	LC379157	LC387351	(Chen et al. 2018b)
*Echinodontium tinctorium*	AFTOL-ID 455	USA	AY854088	AF393056	AY885157	AY864882	AY218482	(Lutzoni et al. 2004)
*Efibula tropica*	WEI 18-149	China	MZ636967	MZ637129	MZ913681	MZ748419	OK136079	(Chen et al. 2021)
*Fomitiporia hartigii*	MUCL 53551	Estonia	JX093789	JX093833	JX093746	–	JX093877	(Amalfi and Decock 2013)
*Fomitopsis pinicola*	AFTOL-ID 770	Unknown	AY854083	AY684164	AY885152	AY864874	AY786056	(Lutzoni et al. 2004)
*Fomitopsis quercina*	FP-56429	USA	KY948809	KY948883	–	KY948989	–	(Justo et al. 2017)
*Fomitopsis serialis*	KHL 12010 ^NT^	Norway	JX109844	JX109844	JX109898	–	JX109870	(Binder et al. 2013)
*Geastrum corollinum*	OSC41996	Unknown	–	DQ218486	DQ219230	–	DQ219052	(Hosaka et al. 2008)
*Geliporus exilisporus*	GC 1702-15	Taiwan	LC378995	LC379153	LC387372	LC379158	LC387352	(Chen et al. 2018b)
*Gloeoporus orientalis*	WEI 16-485	Taiwan	MZ636980	MZ637141	MZ913709	MZ748443	OK136095	(Chen et al. 2021)
*Gloeophyllum sepiarium*	Wilcox-3BB	USA	HM536091	HM536061	HM536110	–	HM536109	(Garcia-Sandoval et al. 2011)
*Gymnopilus picereus*	ZRL2015011	China	LT716066	KY418882	KY419077	KY418980	KY419027	(Zhao et al. 2017)
*Hydnophanerochaete odontoidea*	CWN 00776 ^T^	Taiwan	LC363487	GQ470663	LC387376	LC363498	LC387356	(Wu et al. 2010; Chen et al. 2018b)
*Hydnophlebia aurantia*	WEI 18-623 ^T^	Taiwan	MZ636982	MZ637143	MZ913719	MZ748459	OK136066	(Chen et al. 2021)
*Hyphodermella rosae*	GC 1608-2	Japan	MZ636987	MZ637148	MZ913592	MZ748411	OK135983	(Chen et al. 2021)
*Inonotus griseus*	Dai 13436	China	KX364802	KX364823	MF977775	KX364871	KX364919	(Bian and Dai 2017; Zhu et al. 2019)
*Irpex flavus*	Wu 0705-1	China	MZ636988	MZ637149	MZ913683	MZ748432	OK136087	(Chen et al. 2021)
*Jaapia argillacea*	CBS 252.74	Netherlands	GU187524	GU187581	GU187711	GU187463	GU187788	(Binder et al. 2010)
*Lactarius deceptivus*	AFTOL-ID 682	USA	AY854089	AY631899	AY885158	AY864883	AY803749	(Matheny et al. 2006)
*Lepiota cristata*	ZRL20151133	China	LT716026	KY418841	KY419048	KY418963	KY418992	(Zhao et al. 2017)
*Leptoporus mollis*	RLG-7163-Sp	USA	KY948794	MZ637155	MZ913693	KY948956	OK136101	(Justo et al. 2017; Chen et al. 2021)
*Leptosporomyces raunkiaeri*	HHB-7628	USA	GU187528	GU187588	GU187719	GU187471	GU187791	(Binder et al. 2010)
*Lopharia cinerascens*	FP-105043-Sp	USA	JN165019	JN164813	–	JN164840	JN164874	(Justo and Hibbett 2011)
*Luteoporia albomarginata*	GC 1702-1	Taiwan	LC379003	LC379155	LC387377	LC379160	LC387358	(Chen et al. 2018b)
*Mutatoderma mutatum*	HHB-15479-Sp	USA	KP135296	KP135221	–	KP134870	KP134967	(Floudas and Hibbett 2015)
*Mycoacia fuscoatra*	GC 1705-1	Taiwan	MZ637004	MZ637165	MZ913652	MZ748447	OK136043	(Chen et al. 2021)
*Mycoaciella efibulata*	WEI 16-167	Taiwan	MZ637010	MZ637170	MZ913657	MZ748468	OK136051	(Chen et al. 2021)
*Mycoaciella uda*	FP-101544-Sp	USA	KP135361	KP135232	MZ913649	KP134859	KP134909	(Floudas and Hibbett 2015; Chen et al. 2021)
*Obba rivulosa*	FP-135416-Sp	USA	KP135309	KP135208	–	KP134878	KP134962	(Floudas and Hibbett 2015)
*Odontoefibula orientali*s	GC 1703-76	Taiwan	LC379004	LC379156	LC387379	LC379161	LC387360	(Chen et al. 2018b)
*Onnia tomentosa*	Dai 23683	USA	OM677243	OM677250	OM800826	ON007279	OM937017	(Zhao et al. 2022)
*Phaeophlebiopsis himalayensis*	Chen 3143	Taiwan	MZ637013	MZ637174	MZ913633	MZ748359	OK135992	(Chen et al. 2021)
*Phanerina mellea*	WEI 17-224	Taiwan	LC387333	LC387340	LC387382	LC387345	LC387363	(Chen et al. 2018b)
*Phanerochaete albida*	GC 1407-14	Taiwan	MZ422788	MZ637179	MZ913704	MZ748384	OK136013	(Chen et al. 2021)
*P. albocremea*	CLZhao 32035	China	PQ454011	PQ454677	–	–	–	(Xu et al. 2025)
	**KUC20160609-01**	**Korea**	PX343514	PX343527	**PZ262438**	–	**PZ262487**	(Cho et al. 2026; **This study**)
*P. alnea*	FP-151125	USA	KP135177	MZ637181	MZ913641	MZ748385	OK136014	(Floudas and Hibbett 2015; Chen et al. 2021)
*P. alpina*	Wu 1308-61 ^T^	China	MZ422790	MZ637182	MZ913598	MZ748394	OK136022	(Chen et al. 2021)
*P. amphibolia* “*P. cumulodentata*”	LE 298935	Russia	KP994359	KP994386	–	–	–	(Volobuev et al. 2015)
*P. argillacea*	Wu 9712-18	Taiwan	–	GQ470656	–	–	–	(Wu et al. 2010)
*P. aurantiobadia*	Ghobad-Nejhad 2288 ^T^	China	KP127068	KP127069	–	–	–	(Ghobad-Nejhad et al. 2015)
*P. australis*	HHB-7105-Sp	USA	KP135081	KP135240	–	–	–	(Floudas and Hibbett 2015)
	**SFC20240924-02**	**Korea**	PX343515	PX343528	**PZ262439**	**PZ262463**	**PZ262488**	(Cho et al. 2026; **This study**)
	**SFC20240924-22**	**Korea**	PX343516	PX343529	–	**PZ262464**	**PZ262489**	(Cho et al. 2026; **This study**)
*P. australosanguinea*	MA-Fungi 91309 ^T^	Chile	MH233926	MH233929	–	–	–	(Phookamsak et al. 2019)
*P. bambusicola*	Wu 0707-2 ^T^	Taiwan	MF399404	MF399395	MZ913594	LC314324	OK136009	(Wu et al. 2018b; Chen et al. 2021)
*P. brunnea*	He 4192	China	MT235658	MT248138	–	–	–	(Liu 2016)
*P. burdsallii*	He 2066 ^T^	USA	MT235690	MT248177	–	–	–	(Xu et al. 2020)
*P. burtii*	HHB-4618-Sp	USA	KP135117	KP135241	–	KP134829	KP134945	(Floudas and Hibbett 2015)
*P. cacao* “*P. krikophora*”	HHB-6736-Sp	USA	MZ422817	MZ637203	MZ913611	MZ748403	OK136034	(Chen et al. 2021)
*P. calotricha*	Vanhanen-382	USA	KP135107	–	–	KP134826	–	(Floudas and Hibbett 2015)
*P. canobrunnea*	TNM: CHWC 1506-17 ^T^	Taiwan	LC412093	LC412102	–	–	–	(Wu et al. 2018a)
*P. canolutea*	Wu 9211-105 ^T^	Taiwan	MZ422795	GQ470641	MZ913640	MZ748387	OK136018	(Wu et al. 2010; Chen et al. 2021)
*P. carnosa*	HHB-9195-Sp	USA	KP135129	KP135242	–	KP134831	KP134946	(Floudas and Hibbett 2015)
*P. castanea*	Dai 24915 ^T^	China	PP960566	PP960569	–	–	–	(Luo et al. 2024)
*P. chrysosporium*	HHB-6251-Sp	USA	KP135094	KP135246	–	KP134842	KP134954	(Floudas and Hibbett 2015)
*P. cinerea*	He 5998 ^T^	China	–	MT248171	–	–	–	(Xu et al. 2020)
*P. citrinoalba*	Dai 26584 ^T^	China	PP779892	PP779887	–	–	–	(Luo et al. 2024)
*P. citrinorhizomorpha*	Dai 26417 ^T^	China	PP779891	PP779886	–	–	–	(Luo et al. 2024)
*P. citrinosanguinea*	FP-105385-Sp	USA	KP135100	KP135234	–	KP134824	KP134941	(Floudas and Hibbett 2015)
*P. concrescens*	Spirin 7322 ^T^	Russia	KP994380	KP994382	–	–	–	(Volobuev et al. 2015)
	**KUC20100701B-03**	**Korea**	**PZ231154**	**PZ231214**	–	**PZ262465**	**PZ262490**	**This study**
	**KUC20100707-19**	**Korea**	**PZ231155**	**PZ231215**	**PZ262440**	**PZ262466**	**PZ262491**	**This study**
	**KUC20120509-07**	**Korea**	**PZ231156**	**PZ231216**	**PZ262441**	**PZ262467**	**PZ262492**	**This study**
	**KUC20120605-02**	**Korea**	**PZ231157**	**PZ231217**	**PZ262442**	**PZ262468**	**PZ262493**	**This study**
	**KUC20120618-05**	**Korea**	**PZ231158**	**PZ231218**	**PZ262443**	**PZ262469**	**PZ262494**	**This study**
	**KUC20120618-29**	**Korea**	**PZ231159**	**PZ231219**	**PZ262444**	**PZ262470**	**PZ262495**	**This study**
	**KUC20120717-45**	**Korea**	**PZ231160**	**PZ231220**	**PZ262445**	**PZ262471**	**PZ262496**	**This study**
	**KUC20130903A-08**	**Korea**	**PZ231161**	**PZ231221**	**PZ262447**	**PZ262473**	**PZ262498**	**This study**
	**SFC20150108-22**	**Korea**	**PZ231162**	**PZ231222**	–	–	–	**This study**
	**SFC20150108-25**	**Korea**	**PZ231163**	**PZ231223**	–	–	–	**This study**
	**SFC20150108-27**	**Korea**	**PZ231164**	**PZ231224**	–	–	–	**This study**
	**SFC20150108-30**	**Korea**	**PZ231165**	**PZ231225**	–	–	–	**This study**
	**SFC20150108-31**	**Korea**	**PZ231166**	**PZ231226**	–	–	–	**This study**
	**SFC20150707-64**	**Korea**	**PZ231167**	**PZ231227**	–	–	–	**This study**
	**SFC20150715-03**	**Korea**	**PZ231168**	**PZ231228**	–	–	–	**This study**
	**SFC20150716-01**	**Korea**	**PZ231169**	**PZ231229**	–	–	–	**This study**
	**SFC20160614-56**	**Korea**	**PZ231170**	**PZ231230**	–	–	–	**This study**
*P. concrescens*	**SFC20170619-03**	**Korea**	**PZ231171**	**PZ231231**	–	–	–	**This study**
	**SFC20170712-22**	**Korea**	**PZ231172**	**PZ231232**	–	–	–	**This study**
	**SFC20180803-17**	**Korea**	**PZ231173**	**PZ231233**	–	–	–	**This study**
	**SFC20190510-10**	**Korea**	**PZ231174**	**PZ231234**	–	–	–	**This study**
	**SFC20190730-23**	**Korea**	**PZ231175**	**PZ231235**	–	–	–	**This study**
	**SFC20230729-19**	**Korea**	**PZ231176**	**PZ231236**	–	–	–	**This study**
	**SFC20230817-03**	**Korea**	**PZ231177**	**PZ231237**	–	–	–	**This study**
	**SFC20230908-12**	**Korea**	**PZ231178**	**PZ231238**	–	–	–	**This study**
	**SFC20240411-05**	**Korea**	**PZ231179**	**PZ231239**	–	–	–	**This study**
	**SFC20240503-52**	**Korea**	**PZ231180**	**PZ231240**	–	–	–	**This study**
	**SFC20240522-08**	**Korea**	**PZ231181**	**PZ231241**	–	–	–	**This study**
	**SFC20240812-19**	**Korea**	**PZ231182**	**PZ231242**	–	–	–	**This study**
	**SFC20240826-60**	**Korea**	**PZ231183**	**PZ231243**	–	–	–	**This study**
	**SFC20240826-91**	**Korea**	**PZ231184**	**PZ231244**	–	–	–	**This study**
	**SFC20240827-83**	**Korea**	**PZ231185**	**PZ231245**	–	–	–	**This study**
	**SFC20240827-117**	**Korea**	**PZ231187**	**PZ231247**	–	–	–	**This study**
	**SFC20240828-01**	**Korea**	**PZ231186**	**PZ231246**	–	–	–	**This study**
	**SFC20240828-29**	**Korea**	**PZ231188**	**PZ231248**	–	–	–	**This study**
	**SFC20240828-57**	**Korea**	**PZ231189**	**PZ231249**	–	–	–	**This study**
	**SFC20240915-12**	**Korea**	**PZ231190**	–	–	–	–	**This study**
*P. concrescens* “*P. sordida*”	**KUC20130808-15**	**Korea**	KJ668491	KJ668344	**PZ262446**	**PZ262472**	**PZ262497**	(Jang et al. 2016; **This study**)
*P. crystallina*	GC 1409-7	Taiwan	MZ422803	MZ637189	MZ913607	MZ748404	OK136033	(Chen et al. 2021)
*P. cystidiata*	GC 1708-358 ^T^	Taiwan	LC412096	LC412101	MZ913599	LC412107	OK136025	(Wu et al. 2018a; Chen et al. 2021)
	KUC20180919-02	Korea	–	PX343530	**–**	**–**	**–**	(Cho et al. 2026)
	KUC20200616-27	Korea	PX343517	–	**–**	**–**	**–**	(Cho et al. 2026)
	**SFC20160920-14**	**Korea**	PX343518	PX343531	**PZ262448**	**–**	**PZ262499**	(Cho et al. 2026; **This study**)
	SFC20180706-48	Korea	PX343519	–	**–**	**–**	**–**	(Cho et al. 2026)
	SFC20230317-15	Korea	PX343520	PX343532	**–**	**–**	**–**	(Cho et al. 2026)
*P. ericina*	HHB-2288	USA	KP135167	KP135247	–	KP134834	KP134950	(Floudas and Hibbett 2015)
*P. fissurata*	CLZhao 35311 ^T^	China	PQ454013	PQ454678	–	–	–	(Xu et al. 2025)
*P. flavissima*	CLZhao 17997 ^T^	China	PV175887	PV203435	–	–	–	(Li et al. 2025b)
*P. fusca*	Wu 1409-161 ^T^	China	LC412098	LC412105	MZ913602	LC412109	OK136028	(Wu et al. 2018a; Chen et al. 2021)
	**KUC20121102-19**	**Korea**	KJ668490	KJ668343	**PZ262449**	**PZ262474**	**PZ262500**	(Jang et al. 2016; **This study**)
	F20080905KCM08	Korea	PX343521	PX343533	–	–	–	(Cho et al. 2026)
*P. fuscomarginata*	RLG-10834-Sp	USA	MZ422806	MZ637192	MZ913603	MZ748396	OK136029	(Chen et al. 2021)
*P. ginnsii*	Wu 9210-22 ^T^	Taiwan	MZ422807	MZ637193	MZ913614	MZ748402	–	(Chen et al. 2021)
*P. granulata*	Wu 9210-57 ^T^	Taiwan	MZ422810	MZ637196	–	MZ748406	OK136035	(Chen et al. 2021)
*P. guangdongensis*	Wu 1809-348 ^T^	China	MZ422813	MZ637199	MZ913609	–	–	(Chen et al. 2021)
*P. hainanensis*	He 3562 ^T^	China	MT235692	MT248179	–	–	–	(Boonmee et al. 2021)
*P. hymenochaetoides*	He 5988 ^T^	China	–	MT248173	–	–	–	(Xu et al. 2020)
*P. incarnata*	WEI 16-078 ^T^	Taiwan	MF399407	MF399398	MZ913608	LC314327	–	(Wu et al. 2018b; Chen et al. 2021)
*P. inflata*	Dai 10376	China	JX623929	JX644062	–	–	–	(Jia et al. 2014)
***P. koreana*** sp. nov.	**KUC20130718-43 ^T^**	**Korea**	**PZ231193**	**PZ231252**	**PZ262450**	**PZ262475**	**PZ262501**	**This study**
	**KUC20130626-06**	**Korea**	**PZ231192**	**PZ231251**	–	–	–	**This study**
	**KUC20150814-03**	**Korea**	**PZ231194**	**PZ231253**	–	–	–	**This study**
	**KUC20220609-20**	**Korea**	**PZ231195**	**PZ231254**	–	–	–	**This study**
	**F20100819LJS49**	**Korea**	**PZ231191**	**PZ231250**	–	–	–	**This study**
	**SFC20150715-13**	**Korea**	**PZ231196**	**PZ231255**	**PZ262451**	**PZ262476**	**PZ262502**	**This study**
	**SFC20200716-36**	**Korea**	**PZ231197**	**PZ231256**	–	**PZ262477**	**PZ262503**	**This study**
*P. laevis*	Wu 0309-40	China	MZ422818	GQ470655	MZ913605	MZ748397	OK136026	(Wu et al. 2010; Chen et al. 2021)
	**KUC20130726-06**	**Korea**	KJ668493	KJ668345	**PZ262452**	**PZ262478**	**PZ262504**	(Jang et al. 2016; **This study**)
*P. leptocystidiata*	He 5853 ^T^	China	MT235685	MT248168	–	–	–	(Xu et al. 2020)
*P. livescens*	GC 1612-11	Taiwan	MZ422819	MZ637204	MZ913597	MZ748383	OK136012	(Chen et al. 2021)
*P. magnoliae*	HHB-9829-Sp	USA	KP135089	KP135237	–	KP134838	KP134955	(Floudas and Hibbett 2015)
*P. martelliana*	MA-Fungi 86616	Spain	KF483006	KF528097	–	–	–	Unpublished
***P. membranacea*** sp. nov.	**SFC20170908-17 ^T^**	**Korea**	**PZ231200**	**PZ231258**	–	–	**PZ262505**	**This study**
	**SFC20150518-15**	**Korea**	**PZ231198**	–	–	–	–	**This study**
	**SFC20160907-14**	**Korea**	**PZ231199**	**PZ231257**	**PZ262453**	–	–	**This study**
	**SFC20180822-10**	**Korea**	**PZ231201**	–	**PZ262454**	–	**PZ262506**	**This study**
*P. metuloidea*	He 2766	China	MT235682	MT248164	–	–	–	(Xu et al. 2020)
*P. minor*	He 3988 ^T^	China	MT235686	MT248170	–	–	–	(Xu et al. 2020)
*P. mopanshanensis*	CLZhao 2357 ^T^	China	OR096190	OR461450	OR541927	OR683153	OR733287	(Dong et al. 2024)
*P. ochraceofulva*	KHL 15178	Spain	PV197706	PV197706	–	–	–	(Larsson et al. 2025)
*P. parmastoi*	Wu 880313-6 ^T^	Taiwan	MZ422823	GQ470654	MZ913612	MZ748395	OK136027	(Wu et al. 2010; Chen et al. 2021)
*P. parmastoi*	**SFC20240924-32**	**Korea**	PX343522	PX343534	**PZ262455**	**PZ262479**	**PZ262507**	(Cho et al. 2026; **This study**)
*P. porostereoides*	He 1908 ^T^	China	KX212218	KX212222	–	–	–	(Liu 2016)
	**SFC20140313-05**	**Korea**	PX343523	PX343535	**PZ262456**	–	**PZ262508**	(Cho et al. 2026; **This study**)
*P. pruinosa*	CLZhao 7113 ^T^	China	MZ435347	MZ435351	–	–	–	(Wang and Zhao 2021)
*P. pseudomagnoliae*	PP25 ^T^	South Africa	KP135091	KP135250	–	KP134839	KP134956	(Floudas and Hibbett 2015)
*P. pseudosanguinea*	FD-244 ^T^	USA	KP135098	KP135251	–	KP134827	KP134942	(Floudas and Hibbett 2015)
*P. punctata*	CLZhao 30512 ^T^	China	PQ454016	PQ454681	–	–	–	(Xu et al. 2025)
*P. rhizomorpha*	GC 1708-335 ^T^	Taiwan	MZ422824	MZ637208	–	–	–	(Chen et al. 2021)
	**KUC20121019-22**	**Korea**	KJ668489	KJ668342	**PZ262457**	**PZ262480**	**PZ262509**	(Jang et al. 2016; **This study**)
	**SFC20170821-21**	**Korea**	PX343524	PX343536	**PZ262458**	**PZ262481**	**PZ262510**	(Cho et al. 2026; **This study**)
	SFC20170822-23	Korea	PX343525	–	–	–	–	(Cho et al. 2026)
*P. rhodella*	FD-18	USA	KP135187	KP135258	–	KP134832	KP134948	(Floudas and Hibbett 2015)
*P. robusta*	Wu 1109-69	Taiwan	MF399409	MF399400	–	LC314329	OK136030	(Wu et al. 2018b; Chen et al. 2021)
*P. rufobrunnea* “*P. aculeata*”	Wu 880701-2	Taiwan	MZ422787	GQ470636	MZ913593	MZ748380	OK136008	(Wu et al. 2010; Chen et al. 2021)
*P. sanguineocarnosa*	FD-359	USA	KP135122	KP135245	–	KP134828	KP134944	(Floudas and Hibbett 2015)
*P. shenghuaii*	Dai 24610 ^T^	China	OP874925	OP874920	–	–	–	(Zhang et al. 2023)
	Dai 24609	China	OP874924	OP874919	–	–	–	(Zhang et al. 2023)
*P. sinensis*	He 4660 ^T^	China	MT235688	MT248175	–	–	–	(Xu et al. 2020)
*P. singularis*	He 1873	China	KX212220	KX212224	–	–	–	(Liu 2016)
*P. sordida*	GC 1708-162	China	MZ422828	MZ637212	MZ913637	MZ748388	OK136016	(Chen et al. 2021)
	**KUC20100707-48**	**Korea**	**PZ231202**	**PZ231259**	–	**PZ262482**	**PZ262511**	**This study**
	**KUC20120618-20**	**Korea**	**PZ231203**	**PZ231260**	**PZ262459**	**PZ262483**	**PZ262512**	**This study**
	**KUC20150814-21**	**Korea**	**PZ231204**	**PZ231261**	**PZ262460**	**PZ262484**	**PZ262513**	**This study**
	**SFC20190510-12**	**Korea**	**PZ231205**	**PZ231262**	**PZ262461**	**PZ262485**	**PZ262514**	**This study**
	**SFC20190510-13**	**Korea**	**PZ231206**	**PZ231263**	–	–	**PZ262515**	**This study**
*P. spadicea*	Wu 0504-15 ^T^	China	MZ422837	MZ637219	–	–	–	(Chen et al. 2021)
*P. stereoides*	TNM:F2751 ^T^	Taiwan	MZ422838	MZ637220	MZ913600	MZ748400	OK136032	(Chen et al. 2021)
*P. subcarnosa*	Wu 9310-3 ^T^	Taiwan	MZ422841	GQ470642	MZ913604	MZ748399	OK136024	(Wu et al. 2010; Chen et al. 2021)
	**SFC20180704-27**	**Korea**	PX343526	PX343537	**PZ262462**	**PZ262486**	**–**	(Cho et al. 2026; **This study**)
*P. subceracea*	FP-105974-R	USA	KP135162	KP135255	–	KP134835	KP134951	(Floudas and Hibbett 2015)
*P. subrosea*	He 2421 ^T^	China	MT235687	MT248174	–	–	–	(Xu et al. 2020)
*P. subsanguinea*	CLZhao 10477 ^T^	China	MZ435349	MZ435353	–	–	–	(Wang and Zhao 2021)
*P. subtropica*	CLZhao 8716 ^T^	China	OP605486	OQ195089	–	–	–	(Yu et al. 2023)
*P. subtuberculata*	CLZhao 6838 ^T^	China	OP605485	OQ195087	–	–	–	(Yu et al. 2023)
*P. taiwaniana*	Wu 880824-17 ^T^	Taiwan	MZ422842	GQ470666	MZ913610	MZ748393	OK136021	(Wu et al. 2010; Chen et al. 2021)
	KUC20220714-22	Korea	PQ619348	PQ619378	–	–	–	(Cho et al. 2026)
*P. thailandica*	Wu 1710-3	Vietnam	MZ422843	MZ637223	MZ913601	MZ748401	OK136031	(Chen et al. 2021)
*P. velutina*	GC 1604-56	Taiwan	MZ422844	MZ637224	MZ913642	MZ748386	OK136015	(Chen et al. 2021)
*P. volubilis*	7022526	Russia	PV197708	PV197708				(Larsson et al. 2025)
*P. wuyiensis*	Dai 25530 ^T^	China	PP779888	PP779883	–	–	–	(Luo et al. 2024)
*P. xizangensis*	LWZ20230714-22b ^T^	China	PP959664	PP959651	–	–	–	(Wang et al. 2025b)
*P. yingjiangensis*	CLZhao 37443 ^T^	China	PV820773	–	–	–	–	(Wijesinghe et al. 2025)
*P. yunnanensis*	He 2719 ^T^	China	MT235683	MT248166	–	–	–	(Xu et al. 2020)
*Phanerochaetella angustocystidiata*	Wu 9606-39 ^T^	China	MZ637020	GQ470638	MZ913687	MZ748422	OK136082	(Wu et al. 2010; Chen et al. 2021)
*Phlebia radiata*	AFTOL-ID 484	Unknown	AY854087	AF287885	AY885156	AY864881	AY218502	(Lutzoni et al. 2004)
*Phlebiopsis gigantea*	FCUG 1417	Norway	MZ637051	AF141634	MZ913623	MZ748370	OK135996	(Parmasto and Hallenberg 2000; Chen et al. 2021)
*Podofomes mollis*	RLG-6304-Sp	USA	JN165002	JN164791	JN164901	JN164818	JN164872	(Justo and Hibbett 2011)
*Podoserpula ailaoshanensis*	ZJL2015015 ^T^	China	KU324484	KU324487	KU324494	–	–	(Zhou et al. 2016b)
*Porodaedalea chinensis*	Cui 10252	China	KX673606	MH152358	MG585301	–	MH101479	(Dai 2017)
*Porostereum spadiceum*	Wu 9508-139	China	MZ637062	MZ637263	MZ913697	MZ748445	OK136067	(Chen et al. 2021)
*Ramaria rubella*	AFTOL-ID 724	USA	AY854078	AY645057	AY883435	AY864866	AY786064	(Lutzoni et al. 2004)
*Resiniporus pseudogilvescens*	Wu 1209-46	Taiwan	KY688203	MZ637268	MZ913713	MZ748436	OK136097	(Chen et al. 2018a; Chen et al. 2021)
*Rhizochaete sulphurin*a	HHB-5604	USA	KY273031	GU187610	MZ913707	MZ748363	OK135991	(Binder et al. 2010; Nakasone et al. 2017; Chen et al. 2021)
*Roseograndinia jilinensis*	Wu 1307-137 ^T^	China	MZ637077	MZ637275	MZ913632	MZ748413	OK135985	(Chen et al. 2021)
*Russula emeticicolor*	FH12253	Germany	KT934011	KT933872	–	KT957382	KT933943	(Looney et al. 2016)
*Schenella pityophila*	OSC59743	Unknown	–	DQ218519	DQ219232	–	DQ219057	(Hosaka et al. 2008)
*Scopuloides allantoidea*	WEI 16-060	Taiwan	MZ637081	MZ637279	MZ913664	MZ748463	OK136047	(Chen et al. 2021)
*Serpula himantioides*	MUCL:30528	Belgium	GU187545	GU187600	GU187748	GU187480	GU187808	(Binder et al. 2010)
*Skeletocutis nivea*	ES2008-1	Sweden	JX109858	JX109858	JX109915	–	JX109886	(Binder et al. 2013)
*Steccherinum ochraceum*	KHL 11902	Sweden	JQ031130	JQ031130	JX109893	–	JX109865	(Binder et al. 2013)
*Suillus spraguei*	AFTOL-ID 717	Unknown	AY854069	AY684154	AY883429	AY858965	AY786066	(Shirouzu et al. 2013)
*Terana coerulea*	GC 1507-2	Taiwan	MZ637090	MZ637287	MZ913654	MZ748414	OK136037	(Chen et al. 2021)
*Thelephora ganbajun*	ZRL20151295	China	LT716082	KY418908	KY419093	KY418987	KY419043	(Zhao et al. 2017)
*Trametes versicolor*	FP-135156-Sp	USA	JN164919	JN164809	JN164878	JN164825	JN164850	(Justo and Hibbett 2011)
*Trametopsis cervina*	TJV-93-216T	USA	JN165020	JN164796	JN164882	JN164839	JN164877	(Justo and Hibbett 2011)
*Tyromyces chioneus*	FD-4	USA	KP135311	KP135291	–	KP134891	KP134977	(Floudas and Hibbett 2015)
*Xenasmatella alnicola*	AFTOL-ID 665	USA	DQ411529	AY635768	DQ059052	–	–	Unpublished

^†^Newly-generated sequences in this study are shown in bold.^‡^ Abbreviations: T = Indicate the holotype materials; NT = Indicate the neotype materials.

### Phylogenetic analyses

The reference sequences of ITS, nLSU, *tef1*, *rpb1* and *rpb2* were retrieved from GenBank (Sayers et al. 2025). Sequences were aligned separately for each locus using MAFFT v.7.490 (Katoh and Standley 2013). Alignment, trimming and concatenation were conducted using Geneious Prime 2026.0.2. Phylogenetic analyses were conducted using two concatenated datasets: an ITS+nLSU dataset and a five-locus dataset comprising ITS, nLSU, *tef1*, *rpb1* and *rpb2* sequences. Maximum Likelihood (ML) and Bayesian Inference (BI) analyses were performed for both datasets on the CIPRES web portal (Miller et al. 2015). ML analysis was conducted using RAxML-HPC2 on XSEDE v.8.2.12, using the GTR+G model with 1,000 bootstrap replicates (Stamatakis 2014). The best-fitting substitution model for each locus was determined using jModeltest v.2.1.10. (Darriba et al. 2012). The HKY + I + G model was selected for the ITS and *rpb2* regions, whereas the SYM + I + G model was selected for the nLSU, *tef1* and *rpb1* regions. BI analysis was conducted using MrBayes v.3.2.3 on XSEDE with 20 million generations, sampling every 1,000 generations (Ronquist and Huelsenbeck 2003). Character statistics for the concatenated dataset were calculated using PAUP 4.0b10 under the parsimony criterion (Swofford 2002). The resulting phylogenetic trees were visualised using Figtree v.1.4.3 (Rambaut 2018) and edited in Adobe Illustrator CS6 (Adobe Systems Inc., San Jose, CA, USA). Bootstrap support (BS) values from the ML analysis and posterior probabilities (PP) from the BI analysis are indicated at the corresponding nodes. Values of BS ≥ 70 and PP ≥ 0.70 were considered statistically significant. *Phanerina mellea* (WEI 17-224) was used as the outgroup (Chen et al. 2018b).

### Divergence time estimation

Divergence times were estimated using the Bayesian approach, implemented in BEAST X v.10.5.0, based on a concatenated five-gene dataset (ITS+nLSU+*tef1*+*rpb1*+*rpb2*) (Baele et al. 2025). An XML file was generated using BEAUti v.10.5.0. The HKY nucleotide substitution model was selected as the best-fit model (Hasegawa et al. 1985). An uncorrelated lognormal relaxed clock model was applied to estimate rate variation across branches (Drummond et al. 2006; Lepage et al. 2007) and the Yule speciation process was used as the tree prior (Gernhard 2008).

Two fossil calibrations were applied: *Archaeomarasmius leggetti* Hibbett, D. Grimaldi & Donoghue and *Quatsinoporites cranhamii* S.Y. Sm., Currah & Stockey. *Archaeomarasmius leggetti*, a Cretaceous agaric preserved in amber, was used to constrain the minimum divergence time of the order *Agaricales* (94–90 Mya) (Hibbett et al. 1997), and *Q. cranhamii*, a poroid hymenochaetoid fossil from the Cretaceous, was used to constrain the minimum divergence time of the order *Hymenochaetales* (113 Mya) (Smith et al. 2004). These calibration points have also been adopted in recent divergence time studies of *Polyporales* (Liu et al. 2023a; Zhao et al. 2023; Deng et al. 2025). Calibration priors were assigned using gamma distributions with offsets of 90 Mya for *Agaricales* and 125 Mya for *Hymenochaetales* (Sánchez‐Ramírez et al. 2015). The ucld.mean prior for each gene partition was specified using a gamma distribution (shape = 1.0, scale = 0.001, offset = 0.0). Markov chain Monte Carlo (MCMC) analyses were run for 10 million generations, with sampling every 1,000 generations. The convergence and effective sample sizes (ESS) were assessed using Tracer v.1.7.2. (Rambaut et al. 2018) and all parameters showed ESS values > 200. An ultrametric maximum clade credibility (MCC) tree was generated using TreeAnnotator v.10.5.0 after discarding the first 25% of the sampled trees as burn-in. The final tree with mean node ages and 95% highest posterior density (HPD) intervals was visualised in Figtree v.1.4.3 (Drummond and Rambaut 2007; Rambaut 2018).

### Biogeographic analysis

The historical biogeography of the genus *Phanerochaete* was reconstructed using RASP v.4.2 (Yu et al. 2020). The input phylogenetic tree was inferred in BEAST X v.10.5.0 (Baele et al. 2025), with likelihood calculations accelerated using the BEAGLE library (Ayres et al. 2019). Ancestral areas were estimated using the Bayesian Binary MCMC (BBM) method implemented in the RASP (Yu et al. 2015; Yu et al. 2020). The analysis was run for 1,000,000 MCMC generations with 10 chains, sampled every 100 generations and the first 100 samples were discarded as burn-in. The state frequencies were fixed under the JC model and the amongst-site rate variation was set to equal rates. The probabilities of the ancestral areas were visualised as pie charts at each node of the phylogenetic tree. The geographic distribution was divided into six regions: A, Asia; B, Europe; C, North America; D, South America; E, Africa; and F, Oceania.

## Results

### Phylogeny and diversity of *Phanerochaete*

**Figure 1.**
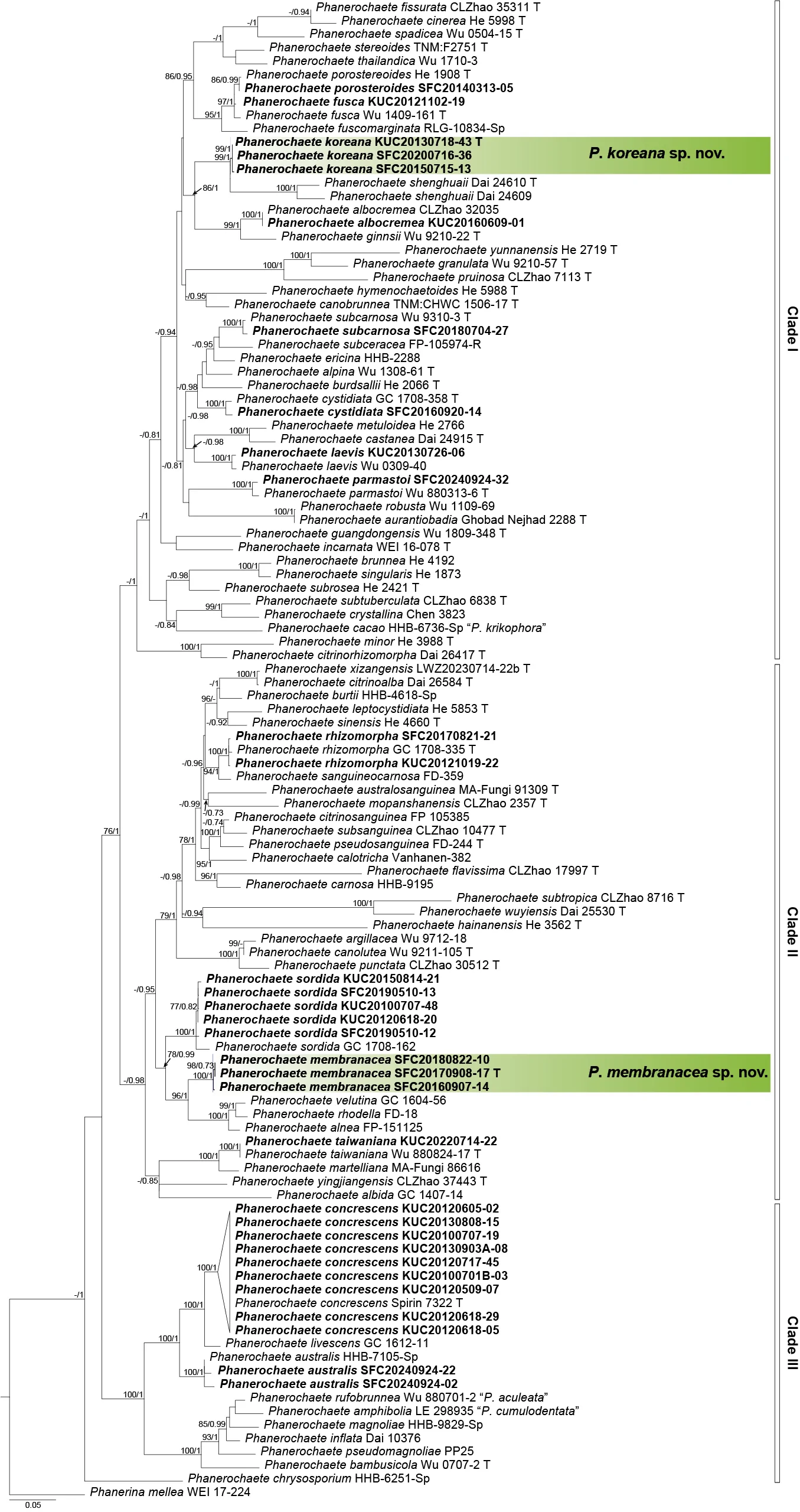
Maximum likelihood phylogeny of *Phanerochaete*, based on concatenated ITS, nLSU, *tef1*, *rpb1* and *rpb2* sequences. Bootstrap support values (BS ≥ 70%) and Bayesian posterior probabilities (PP ≥ 0.7) are shown at the nodes as BS/PP. Newly-generated sequences are shown in bold and newly-described species are highlighted in green boxes. *Phanerina mellea* (WEI 17-224) was used as the outgroup. Detailed information is provided in Table 1. Holotype-derived sequences are indicated with “T”.

ITS and nLSU sequence analyses confirmed that all 72 specimens belonged to *Phanerochaete* (Suppl. material 1). Multi-locus phylogenetic analysis of the ITS, nLSU, *tef1*, *rpb1* and *rpb2* sequences recognised 14 phylogenetically distinct species amongst the examined specimens (Fig. 1). The two novel taxa were recognised, based on morphological comparisons and multi-locus phylogenetic analyses and are described here as *P. koreana* sp. nov. and *P. membranacea* sp. nov. The combined ITS and nLSU dataset comprised 151 taxa and 1,882 aligned characters, of which 1,157 (61.5%) were constant, 267 (14.2%) were variable and parsimony-uninformative and 458 (24.3%) were parsimony-informative. The ML and BI analyses showed similar topologies; thus, only the ML tree is presented. The five-gene dataset included 111 taxa and 4,810 aligned characters, of which 2,801 (58.2%) were constant, 462 (9.6%) were variable and parsimony-uninformative and 1,547 (32.2%) were parsimony-informative. The ML and BI analyses for ITS–nLSU and the five-gene dataset showed similar topologies; thus, only the ML tree is provided (Fig. 1, Suppl. material 1).

The phylogenetic analysis indicated three well-supported clades. Clade I comprised 39 species, including *P. koreana* and *P. laevis*. Clade II comprised 30 species, including *P. membranacea* and *P. sordida*. Clade III comprised 10 species, including *P. australis* and *P. concrescens*. BLAST searches of the ITS and nLSU sequences supported the phylogenetic identification of all Korean specimens included in the phylogenetic analyses. The previously described species shared 99.11%–100.00% ITS sequence identity and 98.12%–100.00% nLSU sequence identity with type-derived or other authoritative reference sequences in GenBank (Suppl. material 3). The two novel species showed lower sequence similarity to their closest species (95.77%–97.58% for ITS and 98.89%–99.22% for nLSU), further supporting their recognition as distinct taxa (Suppl. material 3). For species lacking type-derived sequences (e.g. *P. australis*, *P. laevis* and *P. sordida*), which were described prior to the widespread use of DNA sequencing, sequences from well-documented specimens in previous phylogenetic studies being used as references.

### Divergence time estimation

**Figure 2.**
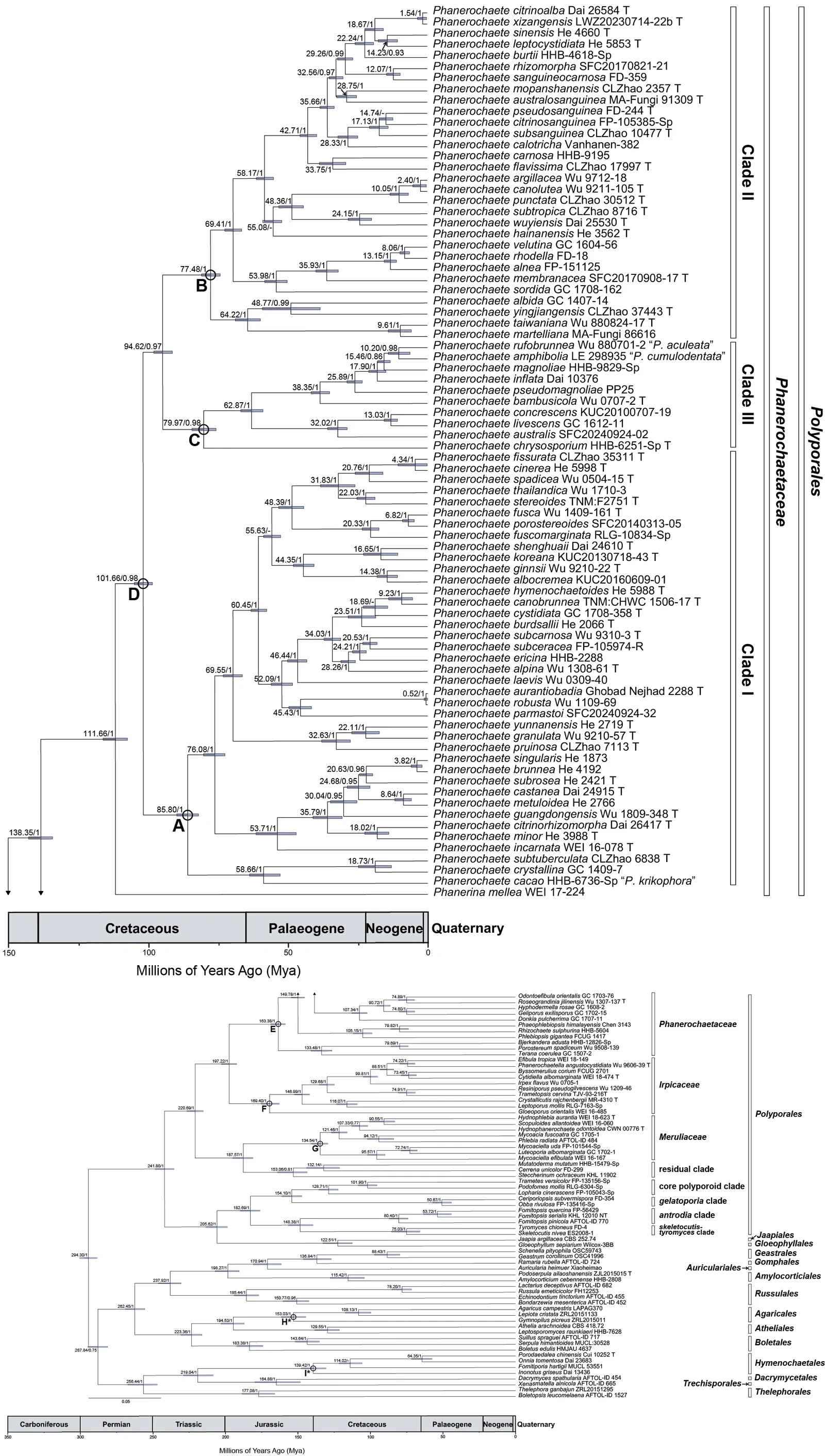
Time-calibrated phylogeny of *Phanerochaete*, based on the concatenated ITS, nLSU, *tef1*, *rpb1* and *rpb2* sequences. Posterior probabilities (PP ≥ 0.8) and the mean divergence times (Mya) are indicated at each node. Horizontal bars represent the 95% highest posterior density (HPD) intervals of node ages. The geological time scale is shown at the bottom of the tree. Holotype and neotype-derived sequences are indicated with “T” and “NT”, respectively.

Divergence time estimation based on the MCC tree, suggested that the crown age of the class *Agaricomycetes* dated back to the Early Permian, at approximately 294.30 Mya (95% HPD: 288.31–300.39 Mya) (Fig. 2). Within the order *Polyporales*, three families (*Irpicaceae*, *Meruliaceae* and *Phanerochaetaceae*) are clustered as the phlebioid clade (Chen et al. 2021). The mean stem age of *Phanerochaetaceae* was estimated at 197.22 Mya (95% HPD: 192.01–202.50 Mya) and the mean crown age was estimated at 163.38 Mya (95% HPD: 158.48–168.54 Mya), corresponding to the Late Jurassic. The divergence times of *Irpicaceae* and *Meruliaceae* were estimated with the mean stem ages of 197.22 Mya (95% HPD: 192.01–202.50 Mya) and 187.57 Mya (95% HPD: 180.88–194.80 Mya) and mean crown ages of 169.40 Mya (95% HPD: 162.78–175.18 Mya) and 134.54 Mya (95% HPD: 129.18–140.71 Mya), respectively (Table 2). These results are consistent with the divergence times of *Irpicaceae* and *Meruliaceae* reported in the previous studies (Wang et al. 2023; Li et al. 2025a).

**Table 2.** Estimated divergence times of the main nodes inferred from BEAST analysis.

**Node**	**Means of stem age (Mya)/95% HPD(Mya)/posterior probabilities**	**Means of crown age (Mya)/95% HPD(Mya)/posterior probabilities**	**Period**
A: *Phanerochaete* Clade I	101.66/98.38–104.86/0.98	85.80/81.94–89.70/1	Late Cretaceous
B: *Phanerochaete* Clade II	94.62/91.15–97.80/0.97	77.48/74.04–80.73/1	Late Cretaceous
C: *Phanerochaete* Clade III	94.62/91.15–97.80/0.97	79.97/75.58–84.17/0.98	Late Cretaceous
D: *Phanerochaete*	111.66/107.33–116.01/1	101.66/98.38–104.86/0.96	Early Cretaceous
E: *Phanerochaetaceae*	197.22/192.01–202.50/1	163.38/158.48–168.54/1	Late Jurassic
F: *Irpicaceae*	197.22/192.01–202.50/1	169.40/162.78–175.18/1	Middle-Late Jurassic
G: *Meruliaceae*	187.57/180.88–194.80/1	134.54/129.18–140.71/1	Early Cretaceous
H*: *Agaricales* (Calibration point)	194.53/186.91–202.98/1	154.03/144.50–161.45/1	Late Jurassic
I*: *Hymenochaetales* (Calibration point)	219.04/208.35–228.88/1	139.42/130.59–147.66/1	Early Cretaceous

The genus *Phanerochaete* was estimated to have originated during the Early Cretaceous, with a mean stem age of 111.66 Mya (95% HPD: 107.33–116.01 Mya) and a mean crown age of 101.66 Mya (95% HPD: 98.38–104.86 Mya). Consistent with the phylogenetic analyses, three major clades were identified: Clade I, II and III. The divergence times of these clades were estimated at mean crown ages of 85.80 Mya (95% HPD 81.94–89.70 Mya), 77.48 Mya (95% HPD: 74.04–80.73 Mya) and 79.97 Mya (95% HPD: 75.58–84.17 Mya), respectively. *Phanerochaete chrysosporium* was the first diverged species. These results indicate that the major lineages of *Phanerochaete* originated during the Late Cretaceous. Subsequent species-level diversification within these clades occurred mainly from the Palaeogene to the Neogene (Table 3).

**Table 3.** Estimated divergence times of *Phanerochaete* species inferred from BEAST analysis.

**Species name**	**Mean stem age (Mya)**	**95% HPD (Mya)**	**Posterior probabilities**	**Epoch**	**Period**
*Phanerochaete albida*	48.77	38.26–58.94	0.99	Eocene	Palaeogene
*P. albocremea*	14.38	10.69–17.99	1	Miocene	Neogene
*P. alnea*	13.15	11.01–15.36	1	Miocene	Neogene
*P. alpina*	28.26	25.76–30.97	1	Oligocene	Palaeogene
*P. amphibolia*	10.20	6.17–13.95	0.98	Miocene	Neogene
*P. argillacea*	2.40	0.30–5.11	1	Pleistocene	Quaternary
*P. aurantiobadia*	0.52	0.01–1.22	1	Pleistocene	Quaternary
*P. australis*	32.02	28.67–35.70	1	Oligocene	Palaeogene
*P. australosanguinea*	28.75	25.20–32.35	1	Oligocene	Palaeogene
*P. bambusicola*	38.35	34.94–41.55	1	Eocene	Palaeogene
*P. brunnea*	3.82	1.93–5.87	1	Pliocene	Neogene
*P. burdsallii*	23.51	18.49–28.37	1	Oligocene	Palaeogene
*P. burtii*	22.24	19.11–25.75	1	Miocene	Neogene
*P. cacao*	58.66	52.76–63.67	1	Palaeocene	Palaeogene
*P. calotricha*	28.33	24.83–31.89	1	Oligocene	Palaeogene
*P. canobrunnea*	9.23	5.29–13.85	1	Miocene	Neogene
*P. canolutea*	2.40	0.30–5.11	1	Pleistocene	Quaternary
*P. carnosa*	33.75	29.09–38.55	1	Oligocene	Palaeogene
*P. castanea*	8.64	5.79–11.74	1	Miocene	Neogene
*P. chrysosporium*	79.97	75.58–84.17	0.98	Late Cretaceous	Cretaceous
*P. cinerea*	4.34	0.07–10.47	1	Pliocene	Neogene
*P. citrinoalba*	1.54	0.20–3.37	1	Pleistocene	Quaternary
*P. citrinorhizomorpha*	18.02	13.88–22.48	1	Miocene	Neogene
*P. citrinosanguinea*	14.74	12.23–17.18	–	Miocene	Neogene
*P. concrescens*	13.03	10.68–15.39	1	Miocene	Neogene
*P. crystallina*	18.73	12.95–24.73	1	Miocene	Neogene
*P. cystidiata*	18.69	14.23–22.92	–	Miocene	Neogene
*P. ericina*	24.21	21.88–26.81	1	Oligocene	Palaeogene
*P. fissurata*	4.34	0.07–10.47	1	Pliocene	Neogene
*P. flavissima*	33.75	29.09–38.55	1	Oligocene	Palaeogene
*P. fusca*	6.82	4.70–8.91	1	Miocene	Neogene
*P. fuscomarginata*	20.33	17.34–23.33	1	Miocene	Neogene
*P. ginnsii*	14.38	10.69–17.99	1	Miocene	Neogene
*P. granulata*	22.11	17.23–27.11	1	Miocene	Neogene
*P. guangdongensis*	30.04	25.30–34.72	0.95	Oligocene	Palaeogene
*P. hainanensis*	55.08	52.04–58.80	–	Eocene	Palaeogene
*P. hymenochaetoides*	9.23	5.29–13.85	1	Miocene	Neogene
*P. incarnata*	53.71	47.00–61.35	1	Eocene	Palaeogene
*P. inflata*	17.90	14.84–20.93	1	Miocene	Neogene
*P. koreana*	16.65	10.57–22.82	1	Miocene	Neogene
*P. laevis*	46.44	43.14–49.99	1	Eocene	Palaeogene
*P. leptocystidiata*	14.23	10.58–17.48	0.93	Miocene	Neogene
*P. livescens*	13.03	10.68–15.39	1	Miocene	Neogene
*P. magnoliae*	15.46	13.27–17.74	0.86	Miocene	Neogene
*P. martelliana*	9.61	5.67–13.92	1	Miocene	Neogene
*P. membranacea*	35.93	31.81–39.83	1	Eocene	Palaeogene
*P. metuloidea*	8.64	5.79–11.74	1	Miocene	Neogene
*P. minor*	18.02	13.88–22.48	1	Miocene	Neogene
*P. mopanshanensis*	28.75	25.20–32.35	1	Oligocene	Palaeogene
*P. parmastoi*	45.43	41.49–49.60	1	Eocene	Palaeogene
*P. porostereoides*	6.82	4.70–8.91	1	Miocene	Neogene
*P. pruinosa*	32.63	27.58–37.91	1	Oligocene	Palaeogene
*P. pseudomagnoliae*	25.89	23.29–28.69	1	Oligocene	Palaeogene
*P. pseudosanguinea*	14.74	12.23–17.18	–	Miocene	Neogene
*P. punctata*	10.05	6.72–13.25	1	Miocene	Neogene
*P. rhizomorpha*	12.07	9.61–14.45	1	Miocene	Neogene
*P. rhodella*	8.06	6.33–9.74	1	Miocene	Neogene
*P. robusta*	0.52	0.01–1.22	1	Pleistocene	Quaternary
*P. rufobrunnea*	10.20	6.17–13.95	0.98	Miocene	Neogene
*P. sanguineocarnosa*	12.07	9.61–14.45	1	Miocene	Neogene
*P. shenghuaii*	16.65	10.57–22.82	1	Miocene	Neogene
*P. sinensis*	14.23	10.58–17.48	0.93	Miocene	Neogene
*P. singularis*	3.82	1.93–5.87	1	Pliocene	Neogene
*P. sordida*	53.98	50.06–58.07	1	Eocene	Palaeogene
*P. spadicea*	20.76	15.88–25.79	1	Miocene	Neogene
*P. stereoides*	22.03	18.74–25.18	1	Miocene	Neogene
*P. subcarnosa*	20.53	18.05–23.13	1	Miocene	Neogene
*P. subceracea*	20.53	18.05–23.13	1	Miocene	Neogene
*P. subrosea*	20.63	16.48–24.83	0.96	Miocene	Neogene
*P. subsanguinea*	17.13	13.94–20.62	1	Miocene	Neogene
*P. subtropica*	24.15	19.91–28.28	1	Oligocene	Palaeogene
*P. subtuberculata*	18.73	12.95–24.73	1	Miocene	Neogene
*P. taiwaniana*	9.61	5.67–13.92	1	Miocene	Neogene
*P. thailandica*	22.03	18.74–25.18	1	Miocene	Neogene
*P. velutina*	8.06	6.33–9.74	1	Miocene	Neogene
*P. wuyiensis*	24.15	19.91–28.28	1	Oligocene	Palaeogene
*P. xizangensis*	1.54	0.20–3.37	1	Pleistocene	Quaternary
*P. yingjiangensis*	48.77	38.26–58.94	0.99	Eocene	Palaeogene
*P. yunnanensis*	22.11	17.23–27.11	1	Miocene	Neogene

^†^Hyphen (–) indicates posterior probability (PP) < 0.8.

### Historical biogeography of the genus *Phanerochaete*

**Figure 3.**
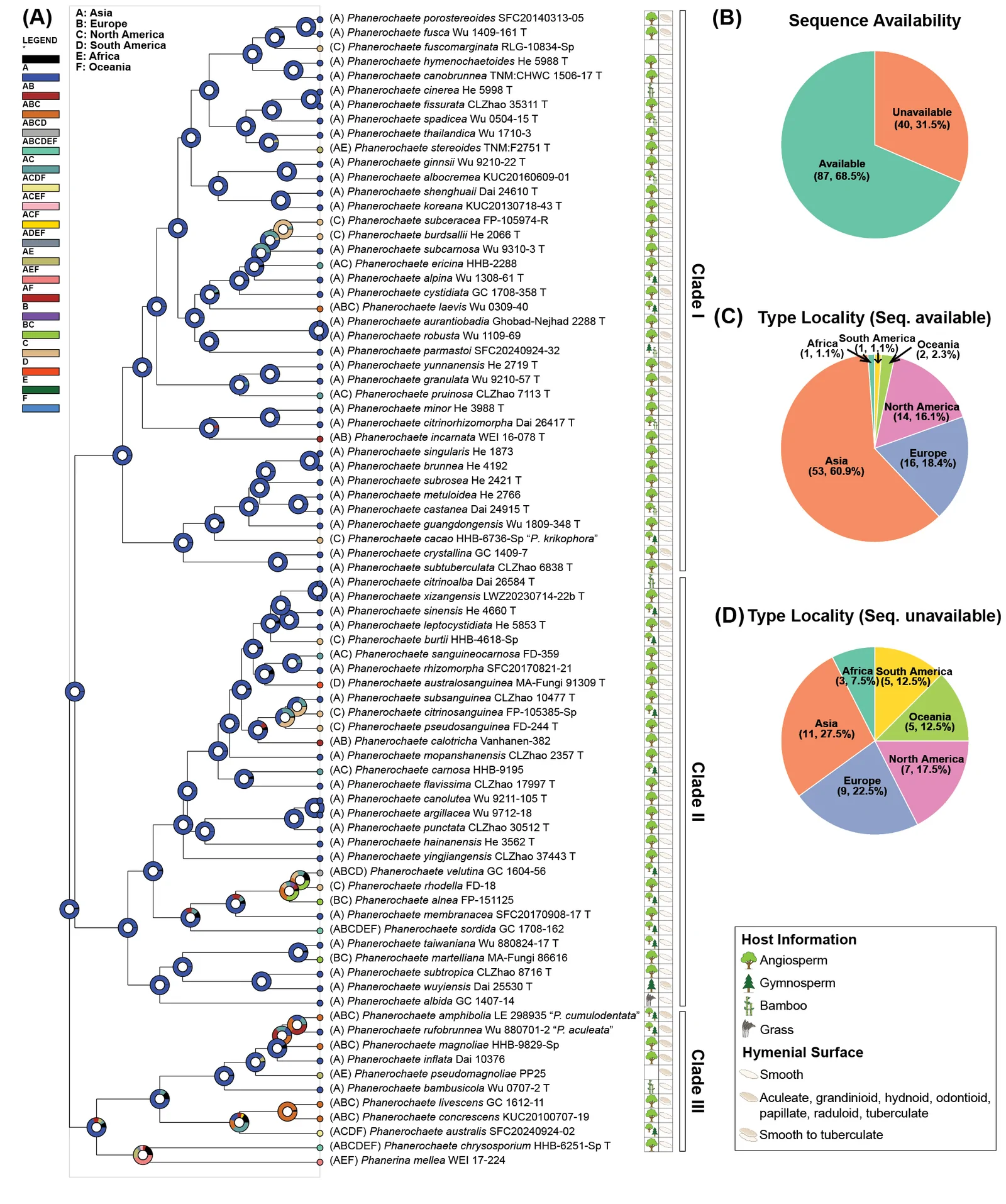
Biogeographic patterns and species characteristics of the genus *Phanerochaete*. **A** Ancestral area reconstruction of the genus *Phanerochaete* inferred using RASP, based on the time-calibrated phylogenetic tree obtained from BEAST analysis. Pie charts at each node represent the relative probabilities of ancestral geographic distributions. Colours correspond to the geographic regions defined in this study. Geographic areas are defined as follows: A = Asia, B = Europe, C = North America, D = South America, E = Africa, F = Oceania. Symbols adjacent to the tree indicate host information and hymenial surface characteristics of each species; **B** Proportion of species with available and unavailable sequence data amongst accepted *Phanerochaete* species; **C** Geographic area of type localities amongst species with available sequence data; **D** Geographic area of type localities amongst species with unavailable sequence data. Holotype-derived sequences are indicated with “T”.

The results of the ancestral area reconstruction, based on RASP analysis, indicated that Asia was the most probable centre of origin for the genus *Phanerochaete* (Fig. 3A). Most internal nodes across the phylogeny showed a high probability of Asian ancestry, suggesting that this genus originated and diversified primarily in Asia. Several dispersal events from Asia to other continents were inferred, particularly in North America and Europe, such as AC (Asia–North America) and AB (Asia–Europe) at several nodes. Dispersal to Africa, Oceania and South America was relatively rare. Several vicariance events between Asia and North America were detected in Clades I and II.

Dispersal patterns differed amongst the three major *Phanerochaete* clades. Clade III exhibited frequent intercontinental dispersal, particularly in *P. amphibolia*, *P. australis*, *P. chrysosporium*, *P. concrescens*, *P. livescens* and *P. magnoliae*. Clades I and II were mainly restricted to Asia. Limited long-distance dispersal was inferred for a few species, including *P. ericina*, *P. incarnata*, *P. laevis*, *P. pruinosa* and *P. stereoides* in Clade I and *P. calotricha*, *P. carnosa*, *P. sanguineocarnosa*, *P. sordida* and *P. velutina* in Clade II. Several species in Clades I and II, including *P. alnea*, *P. burdsallii*, *P. burtii*, *P. cacao*, *P. citrinosanguinea*, *P. fuscomarginata*, *P. martelliana*, *P. pseudosanguinea*, *P. rhodella* and *P. subceracea*, had Asian ancestral lineages, but are now restricted to Europe or North America. This suggests that long-distance dispersal was followed by independent diversification on each continent.

### Host characteristics and morphological features of *Phanerochaete* species

Host characteristics of *Phanerochaete* species were examined, based on available substrate records (Fig. 3A, Table 4). Of the accepted species, 90 species, including two new species, occurred exclusively in angiosperms, 17 occurred in both angiosperms and gymnosperms and six were gymnosperm-specific. The substrates of 11 species were recorded as unknown wood, whereas the other 11 species occurred on other substrates, such as bamboo, shrub and sugarcane.

**Table 4.** Main morphological characteristics, host, and type locality information of accepted *Phanerochaete* species.

**Species**	**Sequence**	**Basidiome**	**Hymenial surface**	**Cystidia**	**Basidiospores (µm)**	**Host**	**Type locality**	**References**
*Phanerochaete affinis*	+	Effused, membranaceous	Pruinose	Subulate, encrusted	Ellipsoid, 4.5–6.0 × 2.5–3.5	Gymnosperm (*Pinus*)	USA	(Burt 1925; Punugu et al. 1980)
*P. albida*	+	Effused, membranaceous	Smooth	Subulate	Ellipsoid, 3.8–4.4 × 1.8–2.3	Grass (Poaceae)	Taiwan	(Wu 1990)
*P. albocremea*	+	Effused, coriaceous	Smooth	Absent	Narrowly ellipsoid, 3.5–5.0 × 2.0–3.0	Angiosperm, Bamboo	China	(Xu et al. 2025)
*P. alnea*	+	Effused, membranaceous	Smooth	Tubular to subfusiform, encrusted	Narrowly ellipsoid to broadly cylindrical, 4.9–7.5 × 2.9–4.0	Angiosperm (*Alnus*, *Betula*, *Fagus*, *Populus*, *Prunus*, *Quercus*, *Salix*, *Sarothamnus*, *Sorbus*), Gymnosperm (*Abies*, *Juniperus*, *Picea*, *Pinus*, *Thuja*)	Finland	(Spirin et al. 2017)
*P. alpina*	+	Effused, membranaceous to coriaceous	Smooth	Subulate to cylindrical, encrusted	Narrowly ellipsoid to cylindrical, 4.6–5.4 × 2.4–3.0	Angiosperm (*Rhododendron*), Gymnosperm (*Pinus*)	China	(Chen et al. 2021)
*P. amphibolia*	+	Effused	Aculeate	Cylindrical	Cylindrical, 4.7–5.9 × 2.4–3.0	Angiosperm (*Corylus*, *Populus*, *Quercus*, *Salix*), Gymnosperm (*Pinus*)	Sweden	(Volobuev et al. 2015; Larsson et al. 2025)
*P. andreae*	–	Effused	Smooth	Absent	Ellipsoid to broadly ellipsoid, 7.5–9.5 × 4.5–6.0	Angiosperm (*Cistus*)	Spain: Canary Islands	(Burdsall Jr et al. 1995)
*P. arenata*	–	Effused, membranaceous to crustaceous	Smooth	Cylindrical, encrusted	Cylindrical to suballantoid, 7.0–11.5 × 3.0–3.5	Angiosperm	South Africa	(Burdsall Jr 1985)
*P. areolata*	–	Effused, membranaceous	Smooth	Cylindrical, encrusted	Oblong ellipsoid, 6.0–8.0 × 3.0–3.5	Unknown	New Zealand	(Cunningham 1963)
*P. argillacea*	+	Effused, ceraceous	Smooth	Cylindrical, encrusted	Ellipsoid, 3.8–5.3 × 2.5–3.0	Angiosperm	Taiwan	(Wu 1998)
*P. arizonica*	+	Effused, crustaceous	Smooth to tuberculate	Cylindrical	Ellipsoid, 5.0–7.0 × 2.5–4.0	Shrub (*Mortonia*)	USA	(Burdsall Jr and Gilbertson 1974; Burdsall Jr 1985)
*P. aurantiobadia*	+	Effused, pruinose	Smooth	Absent	Ellipsoid to subcylindrical, 4.7–6.0 × 2.0–3.0	Angiosperm	China	(Ghobad-Nejhad et al. 2015)
*P. aurata*	–	Effused, crustaceous	Smooth	Cylindrical	Ellipsoid, 7.5–9.0 × 4.5–5.5	Angiosperm	France	(Burdsall Jr 1985)
*P. australis*	+	Effused, membranaceous	Smooth	Subulate to conical, encrusted	Ellipsoid, 3.5–5.0 × 1.8–2.3	Angiosperm (*Casuarina*, *Ficus*, *Psidium*, *Quercus*, *Trema*), Gymnosperm (*Pinus*)	Malaysia: Borneo	(Julich 1980; Gilbertson and Adaskaveg 1993; Floudas and Hibbett 2015; Chen et al. 2021)
*P. australosanguinea*	+	Effused, membranaceous	Smooth	Cylindrical to tapering	Ellipsoid, 4.5–5.5 × 3.0–3.5	Angiosperm (*Nothofagus*)	Chile	(Phookamsak et al. 2019)
*P. avellanea*	+	Effused, membranaceous to crustaceous	Smooth	Absent	Narrowly ellipsoid, 5.0–7.0 × 2.5–3.0	Angiosperm	France	(Burdsall Jr 1985; Bernicchia and Gorjón 2010)
*P. bambusicola*	+	Effused, membranaceous	Smooth	Cylindrical	Narrowly ellipsoid, 6.2–7.8 × 2.5–3.0	Bamboo	Taiwan	(Wu et al. 2018b)
*P. brunnea*	+	Effused, pellicular to membranaceous	Smooth	Absent	Ellipsoid, 4.5–5.5 × 2.3–3.0	Angiosperm	Taiwan	(Wu 1990; Maekawa 1998)
*P. bubalina*	–	Effused, crustaceous	Smooth to odontioid	Absent	Ellipsoid to broadly ellipsoid, 8.5–9.5 × 5.5–6.5	Angiosperm	Spain: Canary Islands	(Burdsall Jr 1985)
*P. burdsallii*	+	Effused, membranaceous	Smooth	Subulate to subcylindrical, encrusted	Ellipsoid, 5.3–6.0 × 2.5–3	Angiosperm (*Populus*)	USA	(Xu et al. 2020)
*P. burtii*	+	Effused, byssoid to fibrous	Smooth	Cylindrical	Ellipsoid to narrowly ellipsoid, 4.5–6.0 × 2.0–2.5	Angiosperm (*Liquidambar*, *Nyssa*, *Thuja*, *Quercus*), Gymnosperm (*Pinus*)	USA	(Burdsall Jr 1975; Floudas and Hibbett 2015)
*P. cacaina*	–	Effused, membranaceous to crustaceous	Smooth	Cylindrical, encrusted	Cylindrical to broadly allantoid, 8.5–12.0 × 3.5–4.5	Gymnosperm (*Pinus*)	France	(Burdsall Jr 1985)
*P. cacao*	+	Effused	Smooth	Subulate, encrusted	Allantoid, 5.0–6.0 × 1.5–2.5	Angiosperm, Gymnosperm (*Pinus*)	Finland	(Eriksson et al. 1978; Larsson et al. 2025)
*P. calotricha*	+	Effused, membranaceous	Smooth	Cylindrical	Narrowly ellipsoid, 4.5–5.5 × 2.0–2.5	Angiosperm (*Castanea*, *Corylus*)	Finland	(Bernicchia and Gorjón 2010; Larsson et al. 2025)
*P. cana*	–	Effused	Grandinioid	Subulate, encrusted	Allantoid, 3.5–4.5 × 1.2–1.5	Unknown	USA	(Burdsall Jr 1985)
*P. canobrunnea*	+	Effused, membranaceous	Smooth	Absent	Ellipsoid to narrowly ellipsoid, 4.2–5.8 × 2.5–3.0	Angiosperm	Taiwan	(Wu et al. 2018a)
*P. canolutea*	+	Effused, subceraceous	Smooth	Absent	Narrowly ellipsoid, 4.7–6.3 × 2.5–3.0	Angiosperm	Taiwan	(Wu 2000)
*P. capitata*	–	Effused, ceraceous	Raduloid	Capitate to cylindrical	Ellipsoid to narrowly ellipsoid, 5.0–6.0 × 2.0–2.8	Angiosperm	Taiwan	(Wu 1998)
*P. carnosa*	+	Effused, membranaceous	Smooth	Subulate, encrusted	Narrowly ellipsoid, 4.5–6.0 × 2.0–3.0	Angiosperm (*Quercus*), Gymnosperm (*Abies*, *Juniperus*, *Larix*, *Picea*, *Pinus*, *Pseudotsuga*)	USA	(Burt 1925; Burdsall Jr 1985)
*P. castanea*	+	Effused, coriaceous	Smooth	Subulate, encrusted	Ellipsoid, 3.9–5.1 × 2.3–3.1	Angiosperm, Bamboo	China	(Luo et al. 2024)
*P. caucasica*	–	Effused, membranaceous to crustaceous	Smooth	Cylindrical, encrusted	Narrowly ellipsoid, 7.0–9.5 × 3.0–3.5	Angiosperm (*Betula*), Gymnosperm (*Pinus*)	Armenia	(Burdsall Jr 1985)
*P. chaparrala*	–	Effused, membranaceous	Smooth	Absent	Broadly ellipsoid, 8.0–10.0 × 6.0–7.5	Angiosperm (*Yucca*)	USA	(Burdsall Jr and Gilbertson 1982)
*P. chrysosporium*	+	Effused, membranaceous	Smooth	Cylindrical	Ovoid, 6.0–7.5 × 3.0–4.0	Angiosperm (*Liriodendron*, *Platanus*, *Ulmus*)	USA	(Burdsall Jr and Eslyn 1974; Burdsall Jr 1985)
*P. cinerea*	+	Effused, membranaceous to coriaceous	Smooth	Absent	Subcylindrical, 4.8–5.6 × 2.0–2.5	Bamboo	China	(Xu et al. 2020)
*P. citri*	–	Effused, membranaceous	Smooth	Absent	Ellipsoid, 5.6–7.0 × 3.5–4.2	Angiosperm (*Citrus*)	India	(De 1991)
*P. citrinoalba*	+	Effused, coriaceous	Smooth	Absent	Ellipsoid to oblong ellipsoid, 4.9–6.3 × 2.4–3.0	Bamboo	China	(Luo et al. 2024)
*P. citrinorhizomorpha*	+	Effused, coriaceous	Smooth	Subulate, encrusted	Ellipsoid, 3.5–4.5 × 1.9–2.8	Angiosperm, Bamboo	China	(Luo et al. 2024)
*P. citrinosanguinea*	+	Effused, membranaceous	Smooth	Cylindrical	Ellipsoid to subcylindrical, 3.5–5.5 × 2.3–3.0	Angiosperm, Gymnosperm	USA	(Floudas and Hibbett 2015)
*P. commixtoides*	–	Effused, cretaceous to membranaceous	Smooth	Cylindrical	Globose to subglobose, 5.0–6.0 × 6.0–7.5	Angiosperm	Taiwan	(Lin and Chen 1990)
*P. concrescens*	+	Effused, pellicular	Smooth	Carrot-shaped, encrusted	Cylindrical, 5.2–6.7 × 2.5–3.1	Angiosperm (*Alnus*, *Betula*, *Corylus*, *Quercus*)	Russia	(Volobuev et al. 2015)
*P. crescentispora*	–	Effused	Smooth	Absent	Crescent-shaped, 7.5–8.5 × 2.0–3.0	Angiosperm (*Myoporum*)	USA: Hawaii	(Gilbertson et al. 2001)
*P. cryptocystidiata*	–	Effused, membranaceous to subceraceous	Smooth	Cylindrical, encrusted	Ellipsoid, 6.5–7.5 × 3.9–4.2	Angiosperm (*Euonymi*)	Germany	(Nakasone 2008)
*P. crystallina*	+	Effused, membranaceous	Smooth to tuberculate	Subfusiform to lanceolate, encrusted	Cylindrical, 5.1–5.7 × 2.2–2.5	Angiosperm	Taiwan	(Chen et al. 2021)
*P. cystidiata*	+	Effused, membranaceous	Smooth to tuberculate	Cylindrical, encrusted	Ellipsoid to narrowly ellipsoid, 4.0–5.3 × 2.5–3.0	Angiosperm	Taiwan	(Wu et al. 2018a)
*P. daliensis*	+	Effused, coriaceous	Grandinioid	Absent	Ellipsoid, elongate to cylindrical, 3.0–6.0 × 1.8–3.0	Angiosperm	China	(Yu et al. 2023)
*P. eburnean*	–	Effused, membranaceous	Smooth	Subulate to conical	Subglobose, 3.7–4.7 × 2.8–3.2	Angiosperm	Taiwan	(Wu 1998)
*P. eichleriana*	–	Unknown	Unknown	Unknown	Unknown	Unknown	Poland	Not available
*P. emplastra*	–	Effused	Smooth	Absent	Unknown	Unknown	Zambia	(Hjortstam 1989)
*P. ericina*	+	Effused, membranaceous	Smooth	Cylindrical, encrusted	Ellipsoid, 5.5–6.5 × 3.0–3.5	Angiosperm	France	(Burdsall Jr 1985)
*P. fissurata*	+	Effused, coriaceous	Smooth	Absent	Ellipsoid, 4–5.5 × 2–3	Angiosperm	China	(Xu et al. 2025)
*P. flavidogrisea*	–	Effused, membranaceous to subceraceous	Smooth	Absent	Broadly ellipsoid, 3.0–3.8 × 2.0–2.8	Angiosperm	Taiwan	(Wu 1998)
*P. flavissima*	+	Effused, leathery	Smooth	Absent	Ellipsoid, 4.5–5.5 × 2.0–3.0	Angiosperm	China	(Li et al. 2025b)
*P. fulva*	–	Effused, membranaceous	Smooth	Subulate, encrusted	Broadly ellipsoid, 4.8–5.8 × 3.0–3.4	Angiosperm	Taiwan	(Wu 1998)
*P. furfuraceovelutinus*	–	Effused	Hydnaceous	Cylindrical	Ellipsoid to subglobose, 3.7–5.0 × 2.5–3.5	Angiosperm, Gymnosperm	Brazil	(Nakasone 2008)
*P. fusca*	+	Effused, membranaceous	Smooth to tuberculate	Cylindrical, encrusted	Narrowly ellipsoid to subcylindrical, 5.7–7.3 × 3.0–3.5	Angiosperm	China	(Wu et al. 2018a)
*P. fuscomarginata*	+	Effused, membranaceous	Smooth	Cylindrical, encrusted	Narrowly ellipsoid, 6.5–8.5 × 3.5–4.5	Unknown	USA	(Burt 1925; Wu 1995)
*P. ginnsii*	+	Effused, subceraceous	Smooth	Absent	Broadly ellipsoid, 4–5 × 2.7–3.0	Angiosperm	Taiwan	(Wu 2000)
*P. globosa*	–	Effused, membranaceous to ceraceous	Smooth	Absent	Globose, subglobose to pyriform, 4.0 × 5.0–6.0	Gymnosperm	Taiwan	(Lin and Chen 1990)
*P. granulata*	+	Effused, membranaceous	Grandinioid	Absent	Ellipsoid to narrowly ellipsoid, 3.7–4.5 × 2.2–2.7	Angiosperm	Taiwan	(Wu 2007)
*P. guangdongensis*	+	Effused, membranaceous to subceraceous	Smooth	Tubular to cylindrical, encrusted	Cylindrical to narrowly cylindrical, 6.2–7.8 × 2.4–3.0	Angiosperm	China	(Chen et al. 2021)
*P. hainanensis*	+	Effused, coriaceous	Smooth	Subulate to subcylindrical	Ellipsoid, 4.5–5.0 × 2.2–2.8	Angiosperm	China	(Boonmee et al. 2021)
*P. hiulca*	+	Effused, crustaceous to membranaceous	Pubescent to fibrillose	Subulate to cylindrical, encrusted	Ellipsoid to narrowly ellipsoid, 5.0–7.0 × 2.5–3.5	Angiosperm	Jamaica	(Burdsall Jr 1985)
*P. hymenochaetoides*	+	Effused, membranaceous to coriaceous	Smooth	Subulate, encrusted	Ellipsoid, 4.0–5.0 × 2.0–2.8	Angiosperm	China	(Xu et al. 2020)
*P. hyphocystidiata*	–	Effused, membranaceous	Smooth	Cylindrical	Ellipsoid to narrowly ellipsoid, 3.8–5.0 × 2.0–2.7	Angiosperm, Grass (*Miscanthus*)	Taiwan	(Wu 1998)
*P. incarnata*	+	Effused, membranaceous	Smooth	Cylindrical	Narrowly ellipsoid to cylindrical, 7.5–9.0 × 3.8–4.5	Angiosperm	Taiwan	(Wu et al. 2018b)
*P. incrustans*	–	Effused, membranaceous	Smooth	Cylindrical, encrusted	Ellipsoid, 6.0–6.5 × 3.0–4.0	Angiosperm (*Citrus*)	Paraguay	(Rajchenberg and Wright 1987)
*P. inflata*	+	Effused, corky	Poroid	Absent	Allantoid to cylindrical, 4.7–5.2 × 2–2.4	Angiosperm	China	(Jia and Cui 2013)
*P. infuscate*	–	Effused	Tuberculate to grandinioid	Subulate, encrusted	Ellipsoid, 4.0–5.0 × 2.5–3.0	Angiosperm	Brazil	(Hjortstam and Ryvarden 2004)
*P. investiens*	–	Effused, pellicular	Hydnoid	Fusiform to subfusiform, encrusted	Ellipsoid, 4–5 × 3.0–3.5	Angiosperm (*Xanthorrhoea*)	Australia	(Hjortstam et al. 2009)
*P. irpicoides*	–	Effused	Raduloid, irpicoid to poroid	Obclavate, subcapitate to subulate	Ellipsoid, 6.0–7.0 × 3.0–3.3	Unknown	Brazil	(Hjortstam 2000)
***P. koreana***	**+**	**Effused, coriaceous**	**Smooth**	**Absent**	**Ellipsoid to cylindrical, 4.2–5.1 × 2.4–3.5**	**Angiosperm (*Quercus*)**	**Korea**	**This study**
*P. laevis*	+	Effused, membranaceous	Smooth	Subcylindrical to subfusiform, encrusted	Narrowly ellipsoid to subcylindrical, 5.0–6.0 × 2.2–2.5	Angiosperm (*Alnus*, *Fagus*, *Genista*), Gymnosperm (*Abies*, *Juniperus*, *Picea*, *Pinus*)	Sweden	(Bernicchia and Gorjón 2010)
*P. leptocystidiata*	+	Effused, pellicular to membranaceous	Smooth to tuberculate	Subulate, encrusted	Ellipsoid to subcylindrical, 5.0–6.0 × 2.5–3.0	Angiosperm	China	(Xu et al. 2020)
*P. livescens*	+	Effused, membranaceous to ceraceous	Smooth to tuberculate	Subulate, carrot-shaped to lageniform, encrusted	Cylindrical, 5.2–7.6 × 2.4–3.3	Angiosperm (*Acer*, *Alnus*, *Caprinus*, *Corylus*, *Fagus*, *Padus*, *Populus*, *Quercus*)	Finland	(Volobuev et al. 2015)
*P. luteoaurantiaca*	+	Effused, membranaceous to ceraceous	Smooth	Subulate to cylindrical, encrusted	Ellipsoid to narrowly ellipsoid, 5.0–5.5 × 2.5–3.0	Unknown	New Zealand	(Burdsall Jr 1985)
*P. magnoliae*	+	Effused, membranaceous	Odontioid to raduloid	Cylindrical, encrusted	Narrowly ellipsoid to subcylindrical, 4.5–6.0 × 2.5–3.0	Angiosperm (*Acacia*, *Castanea*, *Magnolia*, *Quercus*)	USA	(Bernicchia and Gorjón 2010)
*P. martelliana*	+	Effused	Smooth	Subulate	Ellipsoid, 9.0–12.0 × 4.0–5.0	Angiosperm (*Arbutus*, *Castanea*, *Cistus*, *Clematis*, *Erica*, *Juniperus*, *Laurus*, *Quercus*, *Rubus*)	Italy	(Bernicchia and Gorjón 2010)
*P. mauiensis*	–	Effused, soft	Smooth	Cylindrical, conical to clavate, encrusted	Oblong to cylindrical, 5.5–6.0 × 2.5–3.0	Angiosperm (*Eugenia*, *Hibiscus*, *Trema*)	USA: Hawaii	(Gilbertson and Adaskaveg 1993)
***P. membranacea***	**+**	**Effused, membranaceous**	**Smooth**	**Cylindrical to subfusiform, encrusted**	**Narrowly ellipsoid to cylindrical, 5.3–6.2 × 2.8–3.8**	**Angiosperm (*Quercus*)**	**Korea**	**This study**
*P. metuloidea*	+	Effused, membranaceous to coriaceous	Smooth	Subulate to subfusiform, encrusted	Ellipsoid, 5–6 × 2.5–3.0	Angiosperm (*Quercus*)	China	(Xu et al. 2020)
*P. minor*	+	Effused, membranaceous	Smooth	Subcylindrical to subclavate, encrusted	Ellipsoid, 4.0–4.6 × 2.0–2.5	Angiosperm	China	(Xu et al. 2020)
*P. mopanshanensis*	+	Effused, membranaceous to coriaceous	Smooth	Absent	Ellipsoid, 4.2–5.3 × 2.8–3.5	Angiosperm (*Quercus*)	China	(Dong et al. 2024)
*P. ochraceofulva*	+	Effused, ceraceous to subgelatinous	Smooth	Subulate	Ellipsoid to suballantoid, 5.0–6.5 × 3.0–3.5	Angiosperm	France	(Eriksson et al. 1978)
*P. odorata*	–	Effused, membranaceous	Smooth	Ventricose to cylindrical, encrusted	Ovoid to ellipsoid, 7.0–9.0 × 3.5–5.0 µm	Unknown	Sweden	(Boidin and Lanquetin 1987)
*P. oreophila*	–	Effused, membranaceous	odontioid	Clavate to capitate	Cylindrical to narrowly ellipsoid, 5.5–7.0 × 2.5–3.5	Angiosperm (*Metrosideros*)	USA: Hawaii	(Gilbertson and Hemmes 2004)
*P. parmastoi*	+	Effused, membranaceous to subceraceous	Smooth	Subulate, encrusted	Ellipsoid, 5.0–6.2 × 2.5–3.2	Gymnosperm (*Pinus*), Bamboo	Taiwan	(Wu 1990)
*P. parvispora*	–	Effused, membranaceous	Smooth	Absent	Ellipsoid to broadly ellipsoid, 3.2–4.2 × 2.2–2.8	Angiosperm (*Alnus*)	Italy	(Wu and Losi 1995)
*P. porostereoides*	+	Effused to effused-reflexed, coriaceous	Smooth to tuberculate	Absent	Ellipsoid, 4.7–5.3 × 2.5–3.1	Angiosperm	China	(Liu 2016)
*P. pruinosa*	+	Effused, membranaceous to coriaceous	Pruinose	Absent	Cylindrical, 3.5–6.7 × 1.5–2.7	Angiosperm	China	(Wang and Zhao 2021)
*P. pseudomagnoliae*	+	Effused	Hydnaceous	Cylindrical	Ellipsoid, 4.0–6.0 × 2.0–3.0	Unknown	South Africa	(De Koker et al. 2000)
*P. pseudosanguinea*	+	Effused, membranaceous	Smooth	Hyphoid	Ellipsoid to subcylindrical, 4.0–5.5 × 2.0–2.5	Angiosperm	USA	(Floudas and Hibbett 2015)
*P. punctata*	+	Effused, membranaceous	Smooth	Cylindrical to subfusiform	Ellipsoid, 3.5–5.0 × 2.0–3.5	Angiosperm	China	(Xu et al. 2025)
*P. reflexa*	–	Effused, ceraceous	Tuberculate	Absent	Cylindrical to narrowly ellipsoid, 4.8–6.4 × 2.2–2.7	Angiosperm	Taiwan	(Wu 1998)
*P. rhizomorpha*	+	Effused, membranaceous to pellicular	Smooth	Subcapitate to cylindrical	Ellipsoid, 3.9–5.3 × 2.1–3.0	Angiosperm (*Betula*, *Rhododendron*)	Taiwan	(Chen et al. 2021)
*P. rhodella*	+	Effused, velutinous	Smooth	Sword-like, encrusted	Ellipsoid, 5–5.5 × 2.5–3.0	Angiosperm (*Acer*)	USA	(Floudas and Hibbett 2015)
*P. robusta*	+	Effused, membranaceous	Smooth to papillate	Absent	Narrowly ellipsoid to broadly cylindrical, 6.0–7.0 × 2.5–3.0	Angiosperm	Russia	(Burdsall Jr 1985)
*P. rufobrunnea*	+	Effused, membranaceous	Odontioid	Cylindrical to tubular, encrusted	Cylindrical to narrowly ellipsoid, 5.0–6.0 × 2.5–3.0	Angiosperm (*Acacia*), Gymnosperm (*Pinus*)	Iran	(Burdsall Jr 1985)
*P. sacchari*	–	Effused, membranaceous	Smooth	Cylindrical	Ellipsoid, 5.5–7.0 × 3.0–3.5	Sugarcane (*Saccharium*)	Puerto Rico	(Burdsall Jr 1985)
*P. salmoneolutea*	–	Effused, membranaceous to velvety	Smooth	Cylindrical to subulate, encrusted	Ellipsoid, 4.5–6.0 × 2.5–3.0	Angiosperm	USA	(Burdsall Jr and Gilbertson 1974)
*P. sanguineocarnosa*	+	Effused, membranaceous	Smooth	Cylindrical	Subellipsoid to ellipsoid, 4–5.5 × 2.3–3.5	Angiosperm (*Quercus*)	USA	(Floudas and Hibbett 2015)
*P. sandwicensis*	–	Effused, membranaceous	Smooth	Absent	Ellipsoid, 9.0–12.0 × 5.5–7.5	Gymnosperm (*Pinus*)	USA: Hawaii	(Gilbertson and Hemmes 2004)
*P. shenghuaii*	+	Effused, membranaceous to subceraceous	Smooth	Narrowly fusiform to clavate	Ellipsoid, 4.8–6 × 2.5–3.8	Angiosperm	China	(Zhang et al. 2023)
*P. sinensis*	+	Effused, pellicular to membranaceous	Smooth	Subcylindrical	Ellipsoid to subcylindrical, 4.0–5.0 × 2.0–2.5	Angiosperm, Gymnosperm	China	(Xu et al. 2020)
*P. singularis*	+	Effused, membranaceous	Smooth	Clavate	Narrowly ellipsoid to cylindrical, 5.5–7.5 × 2.5–3.5	Angiosperm (*Beilschmiedia*, *Litsea*)	New Zealand	(Burdsall Jr 1985)
*P. sordida*	+	Effused, membranaceous to crustaceous	Smooth	Cylindrical, encrusted	Ellipsoid, 5.0–7.0 × 2.5–3.5	Angiosperm (*Fagus*, *Populus*, *Rhododendron*), Gymnosperm (*Abies*, *Picea*, *Pinus*)	Finland	(Burdsall Jr 1985; Bernicchia and Gorjón 2010; Volobuev et al. 2015)
*P. spadicea*	+	Effused, membranaceous	Smooth	Absent	Cylindrical, 4.2–5.2 × 1.8–2.2	Angiosperm, Bamboo	China	(Chen et al. 2021)
*P. stereoides*	+	Effused, membranaceous	Smooth	Cylindrical	Narrowly ellipsoid, 6.5–8.0 × 3.2–4.0	Angiosperm	Taiwan	(Wu 1995)
*P. suballantoidea*	–	Effused, subceraceous	Smooth to tuberculate	Cylindrical	Suballantoid, 4.7–6.0 × 2.0–2.7	Angiosperm	Taiwan	(Wu 1998)
*P. subcarnosa*	+	Effused, membranaceous	Smooth	Cylindrical to subfusiform	Ellipsoid to cylindrical, 4.9–6.0 × 2.4–2.9	Angiosperm	Taiwan	(Chen et al. 2021)
*P. subceracea*	+	Effused, membranaceous	Smooth	Subulate to subcylindrical, encrusted	Ellipsoid, 4.5–5.5 × 2.0–2.5	Angiosperm	USA	(Burdsall Jr 1985)
*P. subcrassispora*	–	Effused	Smooth	Tubular	Ellipsoid, 4.5–5.0 × 3.0–3.5	Unknown	Australia	(Hjortstam et al. 2009)
*P. subiculosa*	–	Effused, membranaceous	Pubescent to farinaceous	Cylindrical, encrusted	Narrowly ellipsoid, 4.5–5.5 × 2.5–3.0	Gymnosperm	Mexico	(Burdsall Jr 1985)
*P. subquercina*	–	Effused	Odontioid to hydnoid	Absent	Narrowly ellipsoid, 4.0–5.5 × 2.0–3.5	Angiosperm (*Alnus*)	Indonesia: Java	(Hjortstam and Ryvarden 1980)
*P. subrosea*	+	Effused, membranaceous	Smooth	Subcylindrical	Ellipsoid, 5.0–6.0 × 2.5–3.0	Angiosperm	China	(Xu et al. 2020)
*P. subsanguinea*	+	Effused, membranaceous	Smooth	Cylindrical	Narrowly ellipsoid to ellipsoid, 4.5–5.8 × 2.7–3.6	Angiosperm	China	(Wang and Zhao 2021)
*P. subtropica*	+	Effused, coriaceous	Smooth	Fusiform, encrusted	Ellipsoid, 3.4–4.8 × 2.4–3.1	Angiosperm	China	(Yu et al. 2023)
*P. subtuberculata*	+	Effused, coriaceous	Tuberculate	Clavate, encrusted	Ellipsoid, 4.4–6 × 2.5–3.3	Angiosperm	China	(Yu et al. 2023)
*P. taiwaniana*	+	Effused, membranaceous	Smooth	Cylindrical	Ellipsoid, 6.8–7.8 × 3.7–4.3	Angiosperm, Gymnosperm	Taiwan	(Wu 1990)
*P. tamariciphila*	+	Effused, membranaceous	Smooth	Subcylindrical, encrusted	Cylindrical, 7.0–10.0 × 2.8–4.0 µm	Angiosperm (*Tamarix*)	France	(Boidin et al. 1993)
*P. thailandica*	+	Effused, soft	Smooth	Cylindrical to subcapitate	Ellipsoid, 7.0–8.0 × 4.0–4.5	Angiosperm	Thailand	(Sádlíková 2017)
*P. tongbiguanensis*	+	Effused, leathery	Smooth	Subclavate, encrusted	Oblong ellipsoid, 6.0–9.0 × 3.0–4.5	Angiosperm	China	(Deng et al. 2024)
*P. tuberculascens*	–	Effused	Smooth to tuberculate	Absent	Subglobose, 5.5–6.5 × 4.0–4.8	Unknown	Burundi	(Hjortstam 2000)
*P. velutina*	+	Effused, membranaceous	Smooth	Cylindrical to tapered, encrusted	Ellipsoid, 5.0–5.5 × 2.8–3.3	Angiosperm (*Acacia*, *Castanea*, *Fagus*, *Quercus*, *Populus*, *Ulmus*), Gymnosperm (*Abies*, *Picea*, *Pinus*)	Finland	(Burdsall Jr 1985; Bernicchia and Gorjón 2010; Volobuev et al. 2015)
*P. vesiculosa*	–	Effused, subceraceous to crustaceous	Smooth to fibrillose	Clavate to wide fusiform, encrusted	Ellipsoid to broadly ellipsoid, 7.0–9.0 × 4.0–5.0	Angiosperm	Uruguay	(Martínez and Nakasone 2005)
*P. volubilis*	+	Effused, membranaceous to soft-ceraceous	Smooth	Tapering, encrusted	Narrowly cylindrical to cylindrical, 4.1–6.0 × 2.2–3.2	Angiosperm (*Acer*, *Corylus*, *Tilia*), Gymnosperm (*Picea*)	Russia	(Larsson et al. 2025)
*P. wuyiensis*	+	Effused, membranaceous	Smooth to tuberculate	Clavate to fusiform, encrusted	Ellipsoid, 3.6–4.6 × 2.1–3.2	Gymnosperm (*Pinus*)	China	(Luo et al. 2024)
*P. xizangensis*	+	Effused, membranaceous	Smooth	Subcylindrical	Ellipsoid to narrowly ellipsoid, 5.0–6.3 × 2.8–3.2	Angiosperm	China	(Wang et al. 2025b)
*P. yingjiangensis*	+	Effused, membranaceous	Smooth	Absent	Cylindrical, 5.0–7.0 × 3.0–4.0	Angiosperm	China	(Wijesinghe et al. 2025)
*P. yunnanensis*	+	Effused, membranaceous to coriaceous	Grandinioid	Absent	Ellipsoid to subcylindrical, 4.5–6.0 × 2.0–3.0	Angiosperm (*Quercus*)	China	(Xu et al. 2020)

^†^Abbreviations: + = DNA sequence available in GenBank; – = no sequence data.^‡^Newly-described species in this study are shown in bold.

The overall morphological characteristics of *Phanerochaete* species were also summarised (Table 4). Most species possessed effused, membranaceous to coriaceous basidiomata. Basidiospores were generally ellipsoid to cylindrical. In contrast, the characteristics of cystidia varied amongst species, including the presence or absence of cystidia and the occurrence of encrusted or smooth cystidia. These features may be useful for distinguishing species within this genus. Differences in hymenial surface morphology were observed amongst the clades (Fig. 3A). Most species possessed smooth hymenial surfaces, whereas a few species had non-smooth surfaces. In Clade I, 31 species (79.5%) had a smooth hymenial surface, five (12.8%) were smooth to tuberculate and three (7.7%) were non-smooth. In Clade II, 28 species (93.3%) had smooth hymenial surfaces and two (6.7%) were smooth to tuberculate. Clade III showed a relatively high proportion of non-smooth hymenial surfaces: five species (50%) were non-smooth, four (40%) were smooth and one (10%) was smooth to tuberculate.

### Taxonomic key to the *Phanerochaete* species in Korea

**Table:** 

1	Subicular hyphae brown to yellowish-brown	**2**
–	Subicular hyphae hyaline	**3**
2	Hymenial surface tuberculate	***P. fusca***
–	Hymenial surface smooth	***P. porostereoides***
3	Basidiome margin fimbriate, rhizomorphic	**4**
–	Basidiome margin sterile, byssoid	**9**
4	Cystidia absent	***P. koreana***
–	Cystidia present	**5**
5	Cystidia mostly smooth or a few slightly encrusted	**6**
–	Cystidia heavily encrusted with crystals	**7**
6	Basidiome margin rhizomorphic; Hymenial surface smooth	***P. rhizomorpha***
–	Basidiome margin fimbriate; Hymenial surface slightly tuberculate	***P. subcarnosa***
7	Basidiospores ellipsoid to cylindrical, > 3.0 µm in width, Q value > 2.0	***P. laevis***
–	Basidiospores ellipsoid to cylindrical, < 3.0 µm in width, Q value < 2.0	**8**
8	Cystidia > 50 µm in length; Basidiospores 4.4–6.5 × 1.5–2.9 μm	***P. sordida***
–	Cystidia < 50 µm in length; Basidiospores 4.2–5.0 × 2.5–3.0 μm	***P. cystidiata***
9	Cystidia absent	***P. albocremea***
–	Cystidia present	**10**
10	Cystidia mostly smooth or a few slightly encrusted	**11**
–	Cystidia abundantly encrusted with crystals	**12**
11	Hymenial surface coriaceous and smooth; Basidiospores < 6.0 µm in length, < 3.5 µm in width	***P. parmastoi***
–	Hymenial surface subceraceous and velvety; Basidiospores > 6.0 µm in length, > 3.5 µm in width	***P. taiwaniana***
12	Basidiospores narrowly cylindrical, Q value > 2.1; Basidiome margin arachnoid	***P. concrescens***
–	Basidiospores ellipsoid to cylindrical, 1.5 < Q value < 2.2; Basidiome margin byssoid	**13**
13	Hyphae frequently branched	***P. australis***
–	Hyphae unbranched	***P. membranacea***

## Taxonomy

### Phanerochaete koreana

Taxon classificationFungiPolyporalesPhanerochaetaceae

M. Cho & J-J. Kim sp. nov.

4E6D49A4-3664-5E0A-9729-427DF722E63F

863220

Fig. 4.

#### Diagnosis.

Similar to *Phanerochaete albocremea* with white to cream-coloured, coriaceous basidiome and absence of leptocystidia, but distinguished by a sterile margin and smaller basidia.

**Figure 4.**
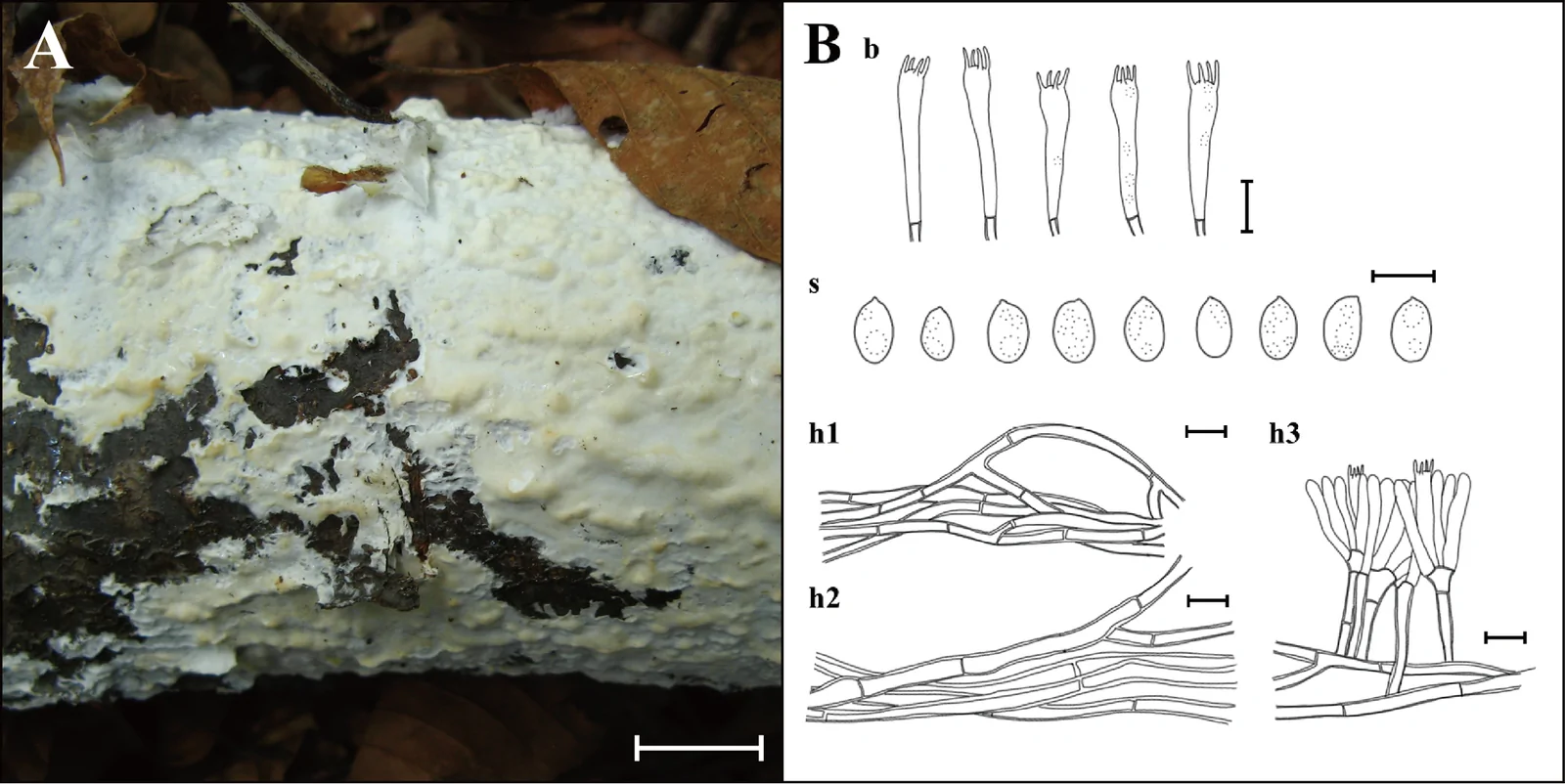
Morphological characteristics of *Phanerochaete koreana* (NIBRFG0000125336, holotype). **A** Basidiome; **B** Microscopic features, **b** basidia; **s** basidiospores; **h1** subhymenial hyphae; **h2** subicular hyphae; **h3** hymenium. Scale bars: 10 mm (**A**); 10 μm (**b, h1–h3**); 5 μm (**s**).

#### Etymology.

“koreana” refers to Republic of Korea, the country where the type locality is situated.

#### Type.

Korea • Gangwon-do, Pyeongchang-gun, Jinbu-myeon, Odaesan National Park 37°46.54'N, 128°34.31'E, ca. 816 m elev., 18 Jul 2013, Y. Jang, S. Jang, H. Lee, (**holotype**: NIBRFG0000125336; **isotype**: KUC20130718-43).

#### Description.

***Basidiomata*** effused, adnate, coriaceous. ***Hymenial surface*** white (2.5YR, 9.5/1) to buff (5Y, 8/4) when young, slightly yellow (10YR, 8/6) when dried, smooth. ***Margin*** rhizomorphic, concolorous with hymenial surface. ***Hyphal system*** monomitic; subhymenial hyphae septate, branched, thick-walled up to 1 μm, without clamp connections, 2.7–4.9 μm in diam., IKI–, CB–, unchanged in KOH. Subicular hyphae septate, sometimes branched, thick-walled up to 1.5 μm, without clamp connections, 3.7–7.5 μm in diam.

***Basidia*** subclavate, 4–spored, smooth, hyaline, thin-walled, bearing several droplets, 26.8–39.8 × 4.5–5.9 μm. ***Leptocystidia*** absent. ***Basidiospores*** ellipsoid to cylindrical, smooth, hyaline, thin-walled, bearing 1–4 droplets, IKI–, CB–, (4.2–)4.6–5.1 × 2.4–3.2(–3.5) μm, L = 4.75 μm, W = 2.90 μm, Q = 1.41–1.93, n = 25.

#### Distribution.

Korea.

#### Habitat.

On dead angiosperm branches, including *Quercus mongolica* in mixed forests.

#### Additional specimens examined.

Korea • Gangwon-do, Chuncheon-si, Dongsan-myeon, Kangwon National University Forest, 37°46.32'N, 127°47.20'E, ca. 362 m elev., 19 Aug 2010, J. S. Lee & Y. Jang, F20100819LJS49 (= NIBRFG0000121915); Korea • Gyeongsangbuk-do, Ulleung-gun, Buk-myeon, Nari basin, 37°30.57'N, 130°52.14'E, ca. 406 m elev., 26 Jun 2013, J.-J. Kim, Y. Jang & S. Jang, KUC20130626-06 (= NIBRFG0000121488); Korea • Gyeonggi-do, Yangpyeong-gun, Yongmun-myeon, Mt. Yongmun, 37°32.55'N, 127°34.21'E, ca. 293 m elev., 14 Aug 2015, J.-J. Kim, KUC20150814-03 (= NIBRFG0000141852); Korea • Jeollanam-do, Hwasun-gun, Mudeungsan National Park, 35°06.23'N, 126°59.16'E, ca. 491 m elev., 09 Jun 2022, M. Cho, S. L. Kwon & S. H. Lee, KUC20220609-20 (= NIBRFG0000513257); Korea • Gyeonggi-do, Goyang-si, Deokyang-gu, Yongdu-dong, Seooreung Royal Tomb, 37°37.28'N, 126°54.06'E, ca. 54 m elev., 15 Jul 2015, J. Y. Park, SFC20150715-13 (= NIBRFG0000141367); Korea • Gangwon-do, Taebaek-si, Sodo-dong, Mt. Hambaek, 37°09.36'N, 128°54.06'E, ca. 1,116 m elev., Y. W. Lim, S. Yoo & Y. Cho, SFC20200716-36 (= NIBRFG0000508309).

#### Notes.

*Phanerochaete koreana* is characterised by a coriaceous, slightly floccose, white to pale basidiomata and it lacks leptocystidia. As most *Phanerochaete* species have leptocystidia, the absence of leptocystidia serves as a key distinguishing feature of this species. Likewise, *P. albocremea*, *P. avellanea*, *P. brunnea*, *P. canobrunnea*, *P. cinerea*, *P. citrinoalba*, *P. fissurata* and *P. spadicea* lack cystidia. However, six out of eight species are macromorphologically distinguished from *P. koreana*, where *P. brunnea*, *P. canobrunnea* and *P. cinerea* have a greyish-brown hymenial surface (Wu 1990; Wu et al. 2018a; Xu et al. 2020). *Phanerochaete avellanea* has yellow to brown hymenial surface (Burdsall Jr 1985) and *P. spadicea* has a brownish hymenial surface (Chen et al. 2021). *Phanerochaete fissurata* has an abundantly cracked basidiome (Xu et al. 2025). Two species, *P. albocremea* and *P. citrinoalba* resemble *P. koreana* in having white to cream-coloured, coriaceous basidiomata. However, *P. albocremea* has a distinctive sterile margin and smaller basidia (16–21 × 4–5.5 μm) (Xu et al. 2025), whereas *P. citrinoalba* has a cracked hymenial surface, smaller basidia (17–25 × 4–7 μm) and larger basidiospores (4.9–6.3 × 2.4–3 μm) (Luo et al. 2024). In the phylogenetic tree, *P. shenghuaii* was most closely related to *P. koreana*. Both have white to yellow basidiomata with rhizomorphic margins. However, *P. shenghuaii* is differentiated by having strongly encrusted subicular hyphae, smaller basidia (22–30 × 4–5 μm) and longer basidiospores (4.8–6 × 2.5–3.8 μm) (Zhang et al. 2023). *Phanerochaete koreana* were collected from the southern (Mudeungsan National Park), central (Goyang-si and Yangpyeong-gun) and northern regions (Odaesan National Park, Chuncheon-si and Taebaek-si) of the Republic of Korea, as well as from Ulleung Island. Its occurrence in relatively temperate areas, such as Mudeungsan and cold areas, including Odaesan and Hambaeksan, indicates that this species is widely distributed throughout the Republic of Korea and is likely to be adapted to a wide range of environmental conditions.

### Phanerochaete membranacea

Taxon classificationFungiPolyporalesPhanerochaetaceae

M. Cho & J-J. Kim sp. nov.

DFDFB389-BDAB-5CBE-820C-D6530093E627

863222

Fig. 5.

#### Diagnosis.

Similar to *Phanerochaete alpina*, which has a pale brown basidiome and heavily encrusted leptocystidia, it is distinguished by more ochraceous basidiomata and smaller basidiospores.

**Figure 5.**
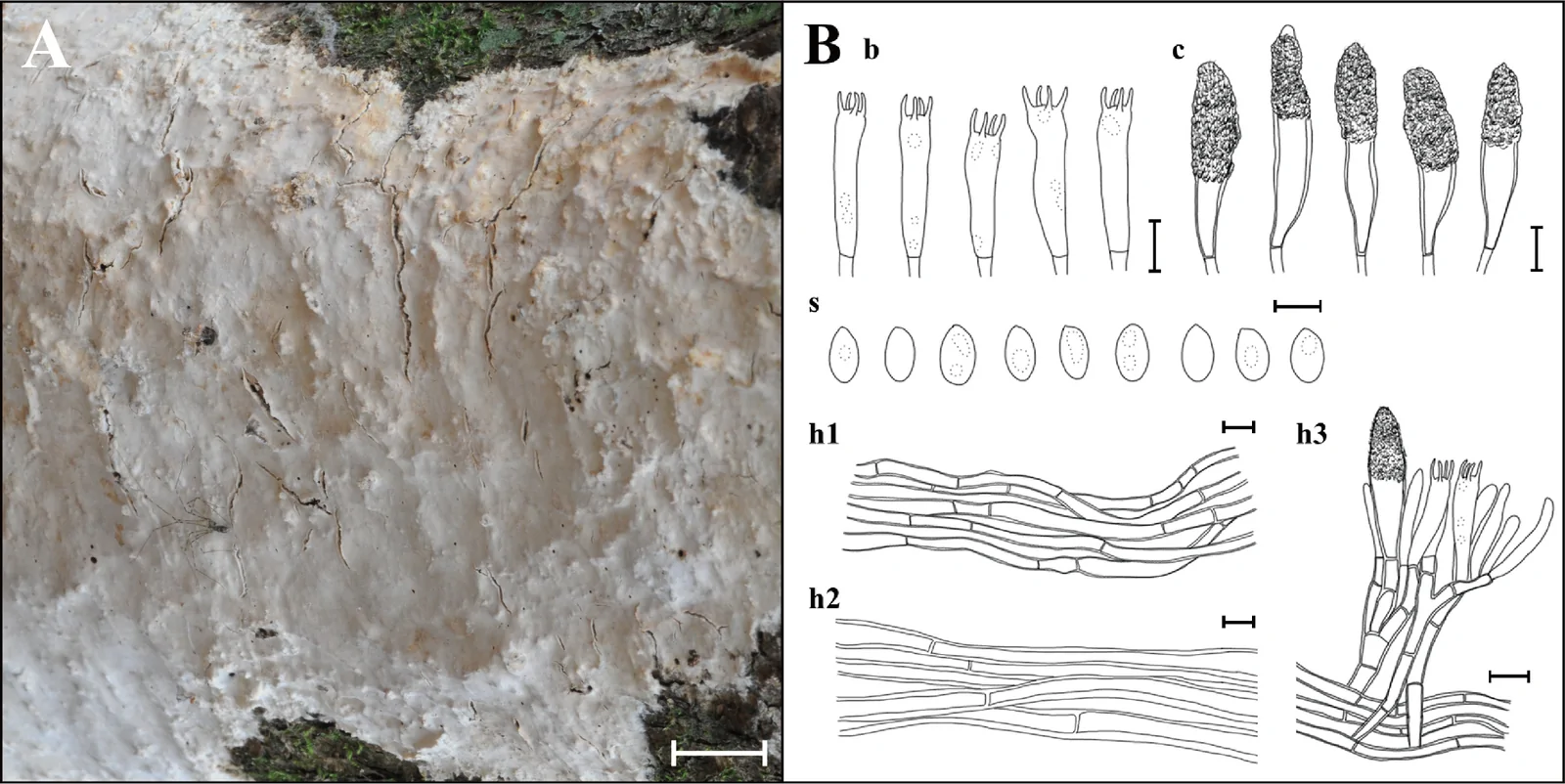
Morphological characteristics of *Phanerochaete membranacea* (NIBRFG0000521710, holotype). **A** Basidiome; **B** Microscopic features, **b** basidia; **c** leptocystidia; **s** basidiospores; **h1** subhymenial hyphae; **h2** subicular hyphae; **h3** hymenium. Scale bars: 10 mm (**A**); 10 μm (**b, h1–h3**); 5 μm (**s**).

#### Etymology.

“membranacea” refers to the membranaceous basidiome of a species.

#### Type.

Korea • Gyeongsangbuk-do, Seongju-gun, Soryun-myeon, Gayasan National Park, 35°48.16'N, 128°08.19'E, ca. 622 m elev., 08 Sep 2017, H. J. Cho, K. H. Park & N. H. Kim, (**holotype**: NIBRFG0000521710; **isotype**: SFC20170908-17).

#### Description.

***Basidiomata*** effused, adnate, membranaceous. ***Hymenial surface*** white (2.5Y, 9.5/2) when young, pale pink (2.5Y, 8.5/2) to pale orange (10YR, 8/4) when dried, smooth, with several cracks when dried. ***Margin*** tuberculate, concolorous with hymenial surface. ***Hyphal system*** monomitic; subhymenial hyphae septate, unbranched, thick-walled up to 1 μm, without clamp connections, 3.6–5.7 μm in diam., IKI–, CB–, unchanged in KOH. Subicular hyphae septate, unbranched, thick-walled up to 2 μm, without clamp connections, 6.5–13.3 μm in diam.

***Basidia*** subclavate, 4–spored, smooth, hyaline, thin-walled, a few bearing droplets, 22.3–29.4 × 5.7–7.5 μm. ***Leptocystidia*** cylindrical to subfusiform, hyaline, thin-walled, abundantly encrusted, 32.6–51.7 × 8.0–9.5 μm. ***Basidiospores*** narrowly ellipsoid to cylindrical, smooth, hyaline, thin-walled, IKI–, CB–, 5.3–6.2 × (2.8–)3.0–3.6(–3.8) μm, L = 5.83 μm, W = 3.37 μm, Q = 1.50–2.09, n = 30.

#### Distribution.

Korea.

#### Habitat.

On dead angiosperm branches, including *Quercus mongolica*, or angiosperm trunks in mixed forests.

#### Additional specimens examined.

Korea • Seoul, Gwanak-gu, Daehak-dong, Mt. Gwanak, 37°27.27'N, 126°56.50'E, ca. 99 m elev., 18 May 2015, J. Y. Park, SFC20150518-15; Korea • Gyeonggi-do, Gwangju-si, Docheok-myeon, Mt. Taehwa, 37°18.41'N, 127°18.40'E, ca. 170 m elev., 07 Sep 2016, J. Y. Park, SFC20160907-14; Korea • Jeollanam-do, Jindo-gun, Uisin-myeon, Mt. Cheomchal, 34°27.56'N, 126°18.40'E, ca. 126 m elev., 22 Aug 2018, J. Y. Park, SFC20180822-10.

#### Notes.

*Phanerochaete membranacea* is characterised by effused and pale brown basidiomata with fimbriate margins and encrusted leptocystidia. *Phanerochaete alpina*, *P. castanea*, *P. citrinorhizomorpha*, *P. livescens* and *P. minor* have heavily encrusted cystidia and fimbriate margins, similar to *P. membranacea*. *Phanerochaete alpina* is distinguished by ochraceous hymenial surface, narrowly cylindrical cystidia (40–60 × 3.5–6 μm) and smaller basidiospores (4.6–5.4 × 2.4–3 μm) (Chen et al. 2021). *Phanerochaete castanea* can be differentiated from *P. membranacea* due to its whitish hymenial surface, thinner cystidia (45–60 × 6–8 μm) and smaller basidiospores (3.7–5.2 × 2.2–3.1 μm) (Luo et al. 2024). *Phanerochaete citrinorhizomorpha* differs in a pinkish hymenial surface, smaller basidia (12–17 × 4–6 μm) and smaller basidiospores (3.3–4.9 × 1.8–3.2 μm) (Luo et al. 2024). *Phanerochaete livescens* is distinguished by carrot-shaped to lageniform cystidia and narrower basidiospores (5.2–7.6 × 2.4–3.3 μm) (Volobuev et al. 2015). *Phanerochaete minor* is distinguished by smaller cystidia (19–37 × 3–7 μm), basidia (15–21 × 3–5 μm), and basidiospores (3.5–4.8 × 2–2.8 μm) (Xu et al. 2020). In the phylogenetic tree, *P. alnea*, *P. rhodella*, *P. sordida* and *P. velutina* were grouped with *P. membranacea* with high BS and PP values. They are morphologically similar to *P. membranacea*, sharing effused, smooth basidiomata and encrusted cystidia. *Phanerochaete alnea* has longer cystidia (52.3–144.7 × 6.2–11 μm) (Spirin et al. 2017) and *P. rhodella* has smaller ellipsoid basidiospores (5–5.5 × 2.5–3.0 μm) (Floudas and Hibbett 2015). *Phanerochaete sordida* has clavate basidia (25–35 × 4.5–5.5 μm) (Burdsall Jr 1985; Bernicchia and Gorjón 2010) and *P. velutina* has longer cystidia (90–150 × 8–12 µm) (Bernicchia and Gorjón 2010). *Phanerochaete alnea*, *P. sordida* and *P. velutina* occur in both angiosperms and gymnosperms (Burdsall Jr 1985; Bernicchia and Gorjón 2010; Volobuev et al. 2015; Spirin et al. 2017), whereas *P. membranacea* has only been observed in angiosperm hosts. Furthermore, *P. alnea* is known from Europe and North America (Spirin et al. 2017) and *P. rhodella* from North America (Floudas and Hibbett 2015), which is geographically distinct from *P. membranacea*.

## Discussion

Consistent with recent taxonomic studies on *Phanerochaete*, the present study supports the utility of ITS and nLSU sequence data for species-level identification in most species of the genus (Xu et al. 2020; Yu et al. 2023; Zhang et al. 2023; Luo et al. 2024; Xu et al. 2025). However, an additional protein-coding marker such as *tef1* is required to delimit closely-related species complexes with limited or polymorphic ITS variation (Spirin et al. 2017; Larsson et al. 2025). Multi-locus datasets comprising four or more genetic markers are widely used in family- or order-level studies (Miettinen et al. 2016; Chen et al. 2021; Li et al. 2025a; Wang et al. 2025a). These studies demonstrated that protein-coding genes, such as *rpb1*, *rpb2* and *tef1*, improve phylogenetic resolution, which is supported by the five-gene analyses in the present study. Floudas and Hibbett (2015) provided the first phylogenetic framework for *Phanerochaete* and related corticioid fungi, focusing on the broader concept of *Phanerochaete* sensu lato. Since then, numerous taxonomic revisions have substantially updated the generic concept of *Phanerochaete*, resulting in the transfer of several species included in their study to other genera. Accordingly, this study provides an updated species-level phylogeny of the currently accepted *Phanerochaete* under the current taxonomic framework. In addition, our analyses suggest that several species currently assigned to *Phanerochaete* (e.g. *P. daliensis*, *P. hiulca*, *P. tamariciphila* and *P. tongbiguanensis*) are more closely related to other genera, highlighting that the generic concept of *Phanerochaete* still requires further analyses. Additional collections and multi-locus sequence data will be necessary to clarify their taxonomic placement.

The estimated divergence times of the major families in *Polyporales*, including *Phanerochaetaceae*, *Irpicaceae* and *Meruliaceae*, were consistent with those of previous studies (Wang et al. 2023; Li et al. 2025a), supporting the robustness of the divergence time estimates in the current study. The diversification pattern of *Phanerochaete* is similar to that observed in other *Polyporales* genera, such as *Ceriporia* (5–63 Mya) and *Meripilus* (6–54 Mya) (Wang et al. 2025a). During this period, major climatic and ecological changes occurred, including the expansion of angiosperms because of warm and humid conditions (Hesheng 1989). Angiosperms emerged in the Late Jurassic and began to expand after a mass extinction event in the Late Cretaceous, replacing gymnosperms (Ramírez-Barahona et al. 2020). The vegetation shift from gymnosperm-to-angiosperm-dominated forests might substantially affect wood-decaying fungi. As many *Phanerochaete* species prefer angiosperm hosts, species diversification after the Late Cretaceous may have been linked to the expansion of angiosperms.

Ancestral area reconstruction suggests that Asia is the centre of origin of *Phanerochaete*, with initial diversification occurring in this region, followed by subsequent dispersal to North America and Europe. The contrasting distribution patterns amongst clades suggest differences in ecological adaptability or dispersal capacity, which are potentially influenced by host, vector, climatic preferences and historical geographic barriers. However, 40 of the 127 accepted species lacked sequence data (Fig. 3B, Table 4). The type localities of species with available sequences were concentrated in Asia, which may introduce geographic sampling bias. Nevertheless, the overall distribution patterns of the type localities were similar between species with and without sequence data (Fig. 3C, D, Table 4), supporting Asia as the most likely centre of origin for *Phanerochaete*. Many species lacking sequence data were described before the widespread use of molecular identification. Thus, future sequencing of these species will enable a more comprehensive evaluation of historical biogeography and ancestral area of *Phanerochaete*.

The host characteristics of *Phanerochaete* species suggest that they prefer angiosperms to gymnosperms and this preference for angiosperms is phylogenetically supported across all clades. Differences in hymenial surface morphology amongst clades may reflect phylogenetic tendencies within the genus. Clade III included a higher proportion of species with non-smooth hymenial surfaces than Clades I and II. However, as the morphology can vary depending on the specimen condition and developmental stage, these morphological differences may represent general tendencies rather than diagnostic keys.

In conclusion, this study provides an updated phylogenetic framework for *Phanerochaete*, based on multi-locus phylogeny, divergence time estimation and historical biogeography, together with the description of two new species from Korea. Our findings improve the current understanding of the evolutionary history of the genus and highlight the importance of multi-locus sequence data for resolving species boundaries and evolutionary relationships. Further studies incorporating additional taxa and molecular data, particularly from non-investigated regions and species lacking sequence data, will further improve the phylogeny and historical biogeography of *Phanerochaete*.

## Supplementary Material

XML Treatment for Phanerochaete koreana

XML Treatment for Phanerochaete membranacea

## Additional information

### Artificial Intelligence (AI) use

AI tools were used solely for English editing and improving grammar and readability. The authors take full responsibility for the content of the manuscript.

### Author ORCIDs

M. Cho https://orcid.org/0009-0007-0834-7294

S.L. Kwon https://orcid.org/0000-0002-2075-5342

Y. Yoo https://orcid.org/0009-0003-2998-4541

S.H. Lee https://orcid.org/0009-0001-2955-6358

C. Kim https://orcid.org/0000-0001-7405-8449

Y.W. Lim https://orcid.org/0000-0003-2864-3449

J.-J. Kim https://orcid.org/0000-0001-8990-2139

### Data availability

All of the data that support the findings of this study are available in the main text or Supplementary Information.

## Supplementary materials

Supplementary material 1 Maximum likelihood (ML) phylogenetic tree of the genus *Phanerochaete*, based on the concatenated ITS and nLSU sequence dataset Data type png https://binary.pensoft.net/file/1750885This dataset is made available under the Open Database License (http://opendatacommons.org/licenses/odbl/1.0/). The Open Database License (ODbL) is a license agreement intended to allow users to freely share, modify, and use this Dataset while maintaining this same freedom for others, provided that the original source and author(s) are credited. Minseo Cho, Sun Lul Kwon, Yeonjae Yoo, Sang Hyun Lee, Changmu Kim, Young Woon Lim, Jae-Jin Kim

Supplementary material 2 PCR amplification conditions used in this study Data type docx https://binary.pensoft.net/file/1750886This dataset is made available under the Open Database License (http://opendatacommons.org/licenses/odbl/1.0/). The Open Database License (ODbL) is a license agreement intended to allow users to freely share, modify, and use this Dataset while maintaining this same freedom for others, provided that the original source and author(s) are credited. Minseo Cho, Sun Lul Kwon, Yeonjae Yoo, Sang Hyun Lee, Changmu Kim, Young Woon Lim, Jae-Jin Kim

Supplementary material 3 BLAST comparison of ITS and nLSU sequences of the examined Korean specimens with type-derived or other authoritative reference sequences Data type docx https://binary.pensoft.net/file/1750887This dataset is made available under the Open Database License (http://opendatacommons.org/licenses/odbl/1.0/). The Open Database License (ODbL) is a license agreement intended to allow users to freely share, modify, and use this Dataset while maintaining this same freedom for others, provided that the original source and author(s) are credited. Minseo Cho, Sun Lul Kwon, Yeonjae Yoo, Sang Hyun Lee, Changmu Kim, Young Woon Lim, Jae-Jin Kim

Supplementary material 4 Concatenated multi-locus (ITS+nLSU+tef1+rpb1+rpb2) sequence alignments of *Phanerochaete* sequence datasets Data type fasta https://binary.pensoft.net/file/1750888This dataset is made available under the Open Database License (http://opendatacommons.org/licenses/odbl/1.0/). The Open Database License (ODbL) is a license agreement intended to allow users to freely share, modify, and use this Dataset while maintaining this same freedom for others, provided that the original source and author(s) are credited. Minseo Cho, Sun Lul Kwon, Yeonjae Yoo, Sang Hyun Lee, Changmu Kim, Young Woon Lim, Jae-Jin Kim

Supplementary material 5 Concatenated ITS and nLSU sequence alignments of *Phanerochaete* sequence datasets Data type fasta https://binary.pensoft.net/file/1750889This dataset is made available under the Open Database License (http://opendatacommons.org/licenses/odbl/1.0/). The Open Database License (ODbL) is a license agreement intended to allow users to freely share, modify, and use this Dataset while maintaining this same freedom for others, provided that the original source and author(s) are credited. Minseo Cho, Sun Lul Kwon, Yeonjae Yoo, Sang Hyun Lee, Changmu Kim, Young Woon Lim, Jae-Jin Kim
