# Supplementary material for: Phylogeny, divergence time and historical biogeography of Phanerochaete (Basidiomycota, Polyporales), with a description of two new species in Korea
Source: IMA Fungus. 2026 Aug 20;17:e204198. doi: 10.3897/imafungus.17.204198 (PMC13522882; doi:10.3897/imafungus.17.204198)
Supplement: Supplementary file 1 [file imafungus-17-e204198-s002.docx]

**Table S1.** PCR amplification conditions used in this study.

| Marker | Initial denaturation | Cycles | Denaturation | Annealing | Extension | Final extension |
| --- | --- | --- | --- | --- | --- | --- |
| ITS, nLSU | 95°C, 5 min | 35 | 95°C, 40 s | 55°C, 40 s | 72°C, 1 min | 72°C, 5 min |
| *tef1* | 96°C, 5 min | 33 | 96°C, 1 min | 45°C, 1 min | 72°C, 1 min | 72°C, 7 min |
| *rpb1*, *rpb2* | 94°C, 2 min | 10 | 94°C, 40 s | 60°C, 40 s | 72°C, 1 min | – |
|  | – | 37 | 94°C, 45 s | 53°C, 1.5 min | 72°C, 2 min | 72°C, 10 min |

^†^Hyphen (–) indicates that the initial denaturation and final extension were performed only once during the two-step PCR protocol.
