# Supplementary material for: Phylogeny, divergence time and historical biogeography of Phanerochaete (Basidiomycota, Polyporales), with a description of two new species in Korea
Source: IMA Fungus. 2026 Aug 20;17:e204198. doi: 10.3897/imafungus.17.204198 (PMC13522882; doi:10.3897/imafungus.17.204198)
Supplement: Supplementary file 2 [file imafungus-17-e204198-s003.docx]

**Table S2.** BLAST comparison of ITS and nLSU sequences of the examined Korean specimens with type-derived or other authoritative reference sequences.

| Species | Korean specimen | Reference sequences (GenBank accession no.) | Type status of reference | ITS identity (%) | nLSU identity (%) |
| --- | --- | --- | --- | --- | --- |
| *P. albocremea* | KUC20160609-01 | *P. albocremea* CLZhao 32235 (PQ454012) | Holotype | 99.84 | – |
|  |  | *P. albocremea* CLZhao 32032 (PQ454676) | Non-type | – | 100.00 |
| *P. australis* | SFC20240924-02 | *P. australis* HHB-7105-Sp (KP135081/KP135240) | Non-type | 100.00 | 100.00 |
|  | SFC20240924-22 | *P. australis* HHB-7105-Sp (KP135081/KP135240) | Non-type | 99.80 | 99.80 |
| *P. concrescens* | KUC20100701B-03 | *P. concrescens* Spirin 7322 (NR_155027/KP994382) | Isotype | 99.68 | 99.11 |
|  | KUC20100707-19 | *P. concrescens* Spirin 7322 (NR_155027/KP994382) | Isotype | 99.84 | 100.00 |
|  | KUC20120509-07 | *P. concrescens* Spirin 7322 (NR_155027/KP994382) | Isotype | 99.84 | 99.63 |
|  | KUC20120605-02 | *P. concrescens* Spirin 7322 (NR_155027/KP994382) | Isotype | 100.00 | 99.53 |
|  | KUC20120618-05 | *P. concrescens* Spirin 7322 (NR_155027/KP994382) | Isotype | 99.75 | 99.63 |
|  | KUC20120618-29 | *P. concrescens* Spirin 7322 (NR_155027/KP994382) | Isotype | 100.00 | 99.63 |
|  | KUC20120717-45 | *P. concrescens* Spirin 7322 (NR_155027/KP994382) | Isotype | 100.00 | 100.00 |
|  | KUC20130808-15 | *P. concrescens* Spirin 7322 (NR_155027/KP994382) | Isotype | 100.00 | 99.83 |
|  | KUC20130903A-08 | *P. concrescens* Spirin 7322 (NR_155027/KP994382) | Isotype | 99.28 | 99.88 |
| *P. cystidiata* | SFC20160920-14 | *P. cystidiata* GC 1708-358 (NR_160572/NG_067799) | Holotype | 99.62 | 99.66 |
| *P. fusca* | KUC20121102-19 | *P. fusca* Wu 1409-161 (NR_160573/NG_067801) | Holotype | 99.81 | 100.00 |
| *P. koreana* | KUC20130718-43 ^T^ | *P. shenghuaii* Dai 24610 (OP874925) | Holotype | 97.12 | – |
|  |  | *P. guangdongensis* Wu 1809-348 (NG_153965) | Holotype | – | 99.11 |
|  | SFC20150715-13 | *P. shenghuaii* Dai 24610 (OP874925) | Holotype | 97.29 | – |
|  |  | *P. guangdongensis* Wu 1809-348 (NG_153965) | Holotype | – | 99.22 |
|  | SFC20200716-36 | *P. shenghuaii* Dai 24610 (OP874925) | Holotype | 97.58 | – |
|  |  | *P. guangdongensis* Wu 1809-348 (NG_153965) | Holotype | – | 99.22 |
| *P. laevis* | KUC20130726-06 | *P. laevis* HHB-17395-Sp (KP135150) | Non-type | 100.00 | – |
|  |  | *P. laevis* KHL 11839 (OR822118) | Non-type | – | 100.00 |
| *P. membranacea* | SFC20170908-17 ^T^ | *P. velutina* HHB-17428-Sp (KP135185) | Non-type | 95.91 | – |
|  |  | *P. velutina* He 3079 (MT248162) | Non-type | – | 98.89 |
|  | SFC20160907-14 | *P. velutina* HHB-17428-Sp (KP135185) | Non-type | 95.77 | – |
|  |  | *P. velutina* He 3079 (MT248162) | Non-type | – | 99.11 |
|  | SFC20180822-10 | *P. velutina* HHB-17428-Sp (KP135185) | Non-type | 95.91 | – |
| *P. parmastoi* | SFC20240924-32 | *P. parmastoi* Wu 880313-6 (NR_175171/NG_153831) | Holotype | 99.46 | 99.89 |
| *P. porostereoides* | SFC20140313-05 | *P. porostereoides* He 1908 (KX212218/KX212222) | Holotype | 99.82 | 100.00 |
| *P. rhizomorpha* | KUC20121019-22 | *P. rhizomorpha* GC 1708-335 (NR_200419/NG_081528) | Holotype | 99.65 | 100.00 |
|  | SFC20170821-21 | *P. rhizomorpha* GC 1708-335 (NR_200419/NG_081528) | Holotype | 99.82 | 100.00 |
| *P. sordida* | KUC20100707-48 | *P. sordida* Wu 1109-55 (MZ422829/MZ637213) | Non-type | 100.00 | 99.78 |
|  | KUC20120618-20 | *P. sordida* Wu 1109-55 (MZ422829/MZ637213) | Non-type | 100.00 | 99.83 |
|  | KUC20150814-21 | *P. sordida* Wu 1109-55 (MZ422829/MZ637213) | Non-type | 98.99 | 99.78 |
|  | SFC20190510-12 | *P. sordida* 20190811-7a (PZ330620) | Non-type | 100.00 | – |
|  |  | *P. sordida* He 5400 (MT248157) | Non-type | – | 100.00 |
|  | SFC20190510-13 | *P. sordida* 20190811-7a (PZ330620) | Non-type | 99.83 | – |
|  |  | *P. sordida* He 5400 (MT248157) | Non-type | – | 100.00 |
| *P. subcarnosa* | SFC20180704-27 | *P. subcarnosa* Wu 9310-3 (NR_175174/GQ470642) | Holotype | 98.73 | 99.77 |
| *P. taiwaniana* | KUC20220714-22 | *P. taiwaniana* Wu 880824-17 (NR_175175/NG_153832) | Holotype | 99.83 | 100.00 |

^†^BLAST searches were performed independently for ITS and nLSU sequences using the closest available type-derived or other authoritative reference sequences in GenBank. Therefore, the reference species may differ between the two genetic markers.

^‡^Hyphen (–) indicates that the reference sequence is available only for the other marker.
